# Mining of Candidate Genes and Developing Molecular Markers Associated with Pokkah Boeng Resistance in Sugarcane (*Saccharum* spp.)

**DOI:** 10.3390/plants13243497

**Published:** 2024-12-14

**Authors:** Haidong Lin, Zhengjie Jiang, Tuan He, Guomeng Li, Mengyu Zhao, Liangyinan Su, Jihan Zhao, Chengwu Zou, Xiping Yang

**Affiliations:** 1 State Key Laboratory for Conservation and Utilization of Subtropical Agro-Bioresources, Guangxi University, Nanning 530005, China; linhaidong9999@gmail.com (H.L.); 2017391014@st.gxu.edu.cn (Z.J.); 18378814393@163.com (T.H.); 2017301025@st.gxu.edu.cn (G.L.); 2017391041@st.gxu.edu.cn (M.Z.); fjnldxzxs@163.com (J.Z.); 2 Guangxi Key Laboratory of Sugarcane Biology & National Demonstration Center for Experimental Plant Science Education, Guangxi University, Nanning 530005, China

**Keywords:** sugarcane, Pokkah Boeng disease, transcriptomes, molecular marker, traditional cultivars

## Abstract

Sugarcane Pokkah Boeng (PB), a fungal disease caused by *Fusarium* spp., poses a significant threat to sugar industries globally. Breeding sugarcane varieties resistant to PB has become a priority, and the mining of PB resistance genes and the development of molecular markers provide a solid foundation for this purpose. This work comprehensively analyzes the genetic components of sugarcane’s resistance to PB using transcriptome sequencing. A segregating population was created by crossing the susceptible parent ROC25 with the resistant parent Yunzhe89-7, which is a traditional cultivar known for its PB resistance. Transcriptome analysis uncovered many differentially expressed genes (DEGs) associated with PB resistance. Utilizing weighted gene co-expression network analysis (WGCNA), we identified gene modules closely related to disease phenotypes. We annotated their functions with bioinformatics tools, particularly focusing on genes enriched in the plant immune response’s MAPK signaling pathway and ABC transporter synthesis pathways. In addition, by integrating whole-genome resequencing data of parental lines and transcriptome data of progeny, we identified a series of putative molecular markers that potentially effectively differentiate between highly resistant and susceptible materials. Our study provides crucial genetic resources and molecular methodologies that are essential for the advancement of sugarcane varieties with improved resistance to PB. These innovations are expected to accelerate the breeding process greatly.

## 1. Introduction

Sugarcane Pokkah Boeng (PB) is caused by *Fusarium* spp., which significantly endangers the global sugarcane industry [1]. The disease was initially recognized and named Pokkah Boeng by Wakker and Went in 1896, and it mostly affected the tip parts and young tissues of sugarcane [2]. During the initial phase of the disease, the base of sugarcane leaves experiences chlorosis and yellowing, with a slightly wrinkled appearance in a corrugated shape. Subsequently, reddish-brown necrotic spots emerge. In severe cases, some sugarcane stems may crack, young heart leaves may become necrotic, and the entire plant may wilt and die. These symptoms result in a significant reduction in both sugarcane yield and sugar content. This disease is considered one of the most major fungal diseases during the elongation period of sugarcane [3,4]. The prevalence of this illness has emerged as a significant threat to the crucial sugarcane-producing regions in China, India, South Africa, and Malaysia, resulting in substantial economic losses [4,5,6]. Specifically, Guangxi, which is the primary region for sugarcane production in China, accounted for over 60% of the country’s total sugar production in 2022 [7]. Since its emergency in the Baise area of Guangxi province, China, in 1982, PB disease has been reported in all sugarcane plantation areas in Guangxi. This becomes particularly serious with the widespread cultivation of susceptible varieties like GT42 and LC05136. As a result, the situation regarding the prevention and control of PB disease has become increasingly severe [8,9]. Currently, there is an urgent need to develop sugarcane varieties that are resistant to PB disease. The identification of genes associated with PB resistance and the creation of molecular markers is crucial for the development of resistant sugarcane cultivars.

Recent advancements in molecular marker technology have significantly enhanced the investigation of genetic diversity and germplasm resources in sugarcane. Daugrois et al. [10] screened and localized the *Bru1* gene, which confers resistance to brown rust in sugarcane. This gene has been demonstrated to provide broad-spectrum resistance against several brown rust isolates. Yang et al. [11] discovered a quantitative trait locus (qORR109) that has a substantial impact (58.0%) on the phenotypic traits associated with resistance to orange rust in the “F_1_” isolate population. They also created a molecular diagnostic marker called G1, which can be used to identify sugarcane plants with resistance to orange rust. This marker is a valuable tool for identifying sugarcane germplasm resources that possess resistance to orange rust. Li et al. [12] utilized the F_1_ generation of the hybrid combination ‘Yuetang 03-393’ × ‘ROC 24’ as the material to distinguish the authenticity of resistant/susceptible to brown rust genetic pools. They screened four Simple Sequence Repeat (SSR) markers, potentially linking to new genes conferring brown rust resistance. This establishes a solid foundation for future efforts in localizing the brown rust resistance genes and breeding sugarcane varieties that are resistant to brown rust. Due to advancements in molecular biology and genomics technology, high-quality genomic sequences have consistently been released for both the ancestors and cultivars of sugarcane. Additionally, there is an increasing availability of published resequencing and transcriptome data for sugarcane cultivars. These resources serve as a foundation for developing molecular markers that can be used to study trait associations in sugarcane.

Several field and greenhouse tests have demonstrated the presence of sugarcane germplasm resources that exhibit significant resistance to sugarcane PB [13,14,15]. This study focused on the segregating population created by crossing ROC25 (♀) and Yunzhe 89-7 (♂). The objective was to identify the resistance genes that are crucial in responding to PB disease. This was achieved by comparing the gene expression patterns among different resistant and susceptible samples utilizing the results of weighted gene co-expression network analysis (WGCNA) and identifying disease-resistance genes belonging to the NBS-LRR gene family. Furthermore, by utilizing whole genome resequencing data from parents and transcriptome data from highly resistant or susceptible materials, we screened variant sites and developed a set of putative molecular markers. This study offers an effective approach and tool for precisely evaluating the genetic composition of sugarcane Pokkah Boeng and identifying advantageous genes.

## 2. Materials and Methods

### 2.1. Sugarcane Resistant and Susceptible Materials

In this study, ROC25, which is susceptible to Pokkah Boeng, was selected as the female parent, and Yunzhe 89-7, which has significant disease resistance characteristics, was chosen as the male parent to construct a hybrid F_1_ generation. These materials were planted in the sugarcane phenotyping nursery in the Guangxi Subtropical Agricultural Science New Base, Guangxi University. The study was conducted over a three-year period during which 151 F_1_ populations, derived from the cross between the female parent ROC25 and the male parent Yunzhe 89-7, were monitored for natural disease incidence. For each material, three replicates were established and randomly distributed across the field. Following a comprehensive evaluation of disease incidence and disease index over these years, 153 materials were selected for further study. This selection includes the two parental lines and 151 F_1_ individuals, which were categorized into the following groups based on their resistance and susceptibility to PB: 17 highly resistant materials (R), 47 moderate-susceptible (healthy) materials (M_H), 47 moderate-susceptible (infected) materials (M_S), 20 highly susceptible (healthy) materials (S_H), and 20 highly susceptible (infected) materials (S_S).

### 2.2. Survey of Phenotypic Data of the Hybrid Population

Referring to the studies of Wang et al. [16] to determine the disease level and disease resistance evaluation criteria, the Pokkah Boeng disease incidence in the naturally occurring population was evaluated in June 2021 based on the results of the phenotypic survey by He et al. [17]. The plants were graded according to the severity of the disease, which was categorized into five grades (Table S1). Disease severity index (DSI) = [∑ (ni × vi)/4N] × 100; n represents the number of samples in the level, v represents the susceptibility level, and N represents the overall samples of the participants. Evaluation of the disease severity index (DSI) of Pokkah Boeng was calculated according to the above method, and the average of three biological replications was taken as the final result (Table S2).

### 2.3. Differential Expression Analysis of Transcriptome Data from Resistant and Susceptible Sugarcane Groups

A total of 153 materials were collected from the above hybrid population, and the transcriptome sequencing was performed in Annoroad Gene Technology Co., Ltd. (Beijing, China) using the sequencing platform DNBSEQ-T7. The sequencing data were uploaded to a local high-performance computing server for subsequent analyses. Quality control of the transcriptome sequencing data was performed using Fastp v.0.23.2 to filter out high-quality reads. The filtered reads were subsequently aligned with the CDS sequences of the sugarcane relatives *S. spontaneum* and *S. officinarum* using Bowtie2 v.2.2.5. The only uniquely aligned reads were quantitatively analyzed by Salmon v.1.4.0 and transcript molecules per million (TPM) values were used to assess gene expression. Further, gene expression differences between different susceptible or resistant materials were statistically analyzed using the edgeR package in R. Based on the analysis results of the edgeR package, genes with FDR (False Discovery Rate) less than 0.05 and |log2 (fold change)| > 1 were selected as differentially expressed genes (DEGs).

### 2.4. Functional Annotation of Differentially Expressed Genes

Kyoto Encyclopedia of Genes and Genomes (KEGG) [18] and Gene ontology (GO) [19] enrichment analyses of DEGs were performed to identify the metabolic pathways and biological functions in which these genes are involved. For annotation, reference genomes were selected: rice, maize, wheat, and Arabidopsis. Download the Swiss-Prot database and TrEMBL database from the protein database Uniprot (https://www.uniprot.org/, accessed on 15 March 2023) and compare all CDS sequences of *S. spontaneum* and *S. officinarum* to the two databases by using Diamond (v0.9.14.115) to obtain annotation information corresponding to the sugarcane genes for GO annotation. Genes enriched to important pathways were queried by NCBI non-redundant protein sequences (Nr https://www.ncbi.nlm.nih.gov/, accessed on 15 March 2023) and NCBI non-redundant nucleotide sequences (Nt https://www.ncbi.nlm.nih.gov/, accessed on 15 March 2023) to determine their functions.

### 2.5. Weighted Gene Co-Expression Network Analysis

Weighted co-expression gene networks were constructed using the WGCNA software package (v1.71.) in R for differentially expressed genes obtained from transcriptome analysis and filtered according to the top 75% of the median absolute deviation (MAD) values with MAD > 0.01. In order to make the gene network conform to the scale-free distribution, the soft thresholding power was set to 3, the minimum number of genes in a module was set to 30, and the distance of merging similarity modules was set to 0.25 to construct the co-expression network. Gene modules with a *P* value less than 0.05 were identified as significantly associated with the phenotypic traits.

### 2.6. Real-Time Fluorescence Quantitative PCR Analysis

To validate the results of transcriptome sequencing, a total of 9 candidate key genes were screened in 5 representative samples for real-time fluorescence quantitative PCR analysis. Primer design was accomplished using Primer-BLAST software (https://www.ncbi.nlm.nih.gov/tools/primer-blast/index.cgi, accessed on 15 March 2023), and the specificity was verified by the Primer Check tool of the TBtools platform. The validated primer sequences were synthesized by Sangon Biotech Co., Ltd. (Shanghai, China). The details of the primer sequences are shown in Table S3. The RT-qPCR was performed using the Light Cycler 96 RT-qPCR System (Roche Diagnostics, Mannheim, Germany). Three replicates were carried out independently for each sample, melting curves were analyzed at the end of the reaction, and the relative expression was calculated using the 2^−ΔΔCT^ algorithm.

### 2.7. Development of Molecular Markers

Whole genome resequencing data of the parents and transcriptome data of the highly resistant/susceptible samples were combined to identify insertion/deletion (InDel) polymorphism loci that could be used to differentiate between highly resistant and highly susceptible materials in the F_1_ generation of sugarcane. Whole genome resequencing data from our laboratory’s ROC25 and Yunzhe 89-7 were utilized, and the sequencing data were quality-controlled by FastP. The genome sequences of *S. spontaneum* and *S. officinarum* were used as references, and the filtered reads were aligned with the reference sequences using BWA (v0.7.17) [20]. After Picard de-weighting, variant detection was performed using the HaplotypeCaller tool of GATK (v4.2.6.1) [21] and the VCF files were merged by the CombineGVCFs tool, SelectVariants to pick the InDels filtered by the VariantFiltration tool to obtain the High-quality variant loci were filtered by QD < 2.0 || FS > 60.0 || MQ < 40.0 || SOR > 3.0 || MQRankSum < −12.5 || ReadPosRankSum < −8.0. In addition, Bam files obtained from transcriptome analysis of the resistance groups were merged by using Samtools software (v1.15.1) [22]. Files were used to form two highly resistant (R1, R2) and two highly susceptible (S1, S2) samples; after removing the duplicates by Picard, the InDel polymorphism loci among the offspring were obtained by using the same method and filtering criteria as those of the parents. The InDel markers identified from both the whole-genome resequencing data of the parents and the transcriptome data of the F_1_ generation have been integrated using Integrative Genomics Viewer (IGV) for visualization.

## 3. Results

### 3.1. Transcriptome Analysis of Sugarcane Challenged by Pokkah Boeng Pathogens

This study thoroughly analyzed the transcriptome of a sugarcane population for resistance and susceptibility to Pokkah Boeng pathogens. The population consisted of two parents (ROC25 as the female and Yunzhe 89-7 as the male) and the hybrid segregating population constructed by them, from which a total of 153 samples were selected as the experimental materials in this study. These samples included the two parental lines, as well as 17 highly resistant progeny (R), 47 moderate-susceptible progeny at healthy state (M_H), and the same 47 moderate-susceptible progeny at infected state (M_S), 20 highly susceptible progeny at healthy state (S_H) and the same 20 highly susceptible progeny at infected state (S_S). A total of 1805.5 Gb of raw data were generated by RNA-Seq technology. After quality control, 1802.3 Gb of clean data were extracted, with a Q20 ratio exceeding 96.0% and GC content ranging from 51.7% to 55.4% (Table S5), indicating that the data quality was suitable for further analysis.

**Figure 1:**
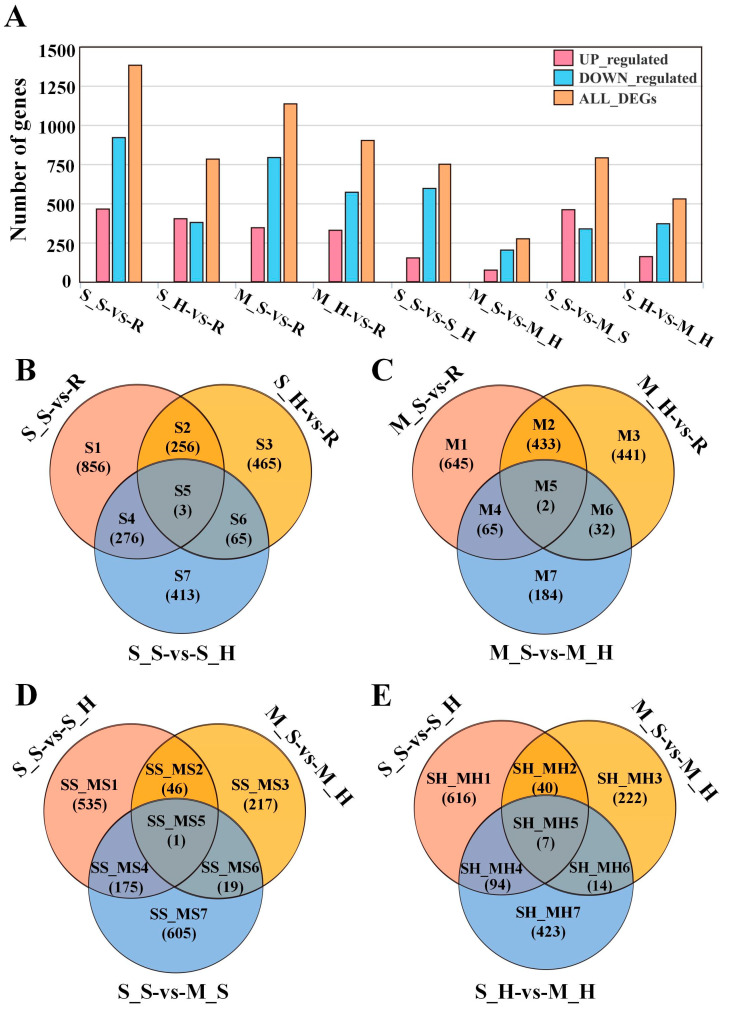
Analysis of DEGs in different groups of sugarcane samples. (**A**) Number of DEGs in each group. (**B**) Venn analysis of the highly resistant and highly susceptible group. (**C**) Venn analysis of the highly resistant and moderate-susceptible group. (**D**) Venn analysis of the highly susceptible (infected) and moderate-susceptible (infected) group. (**E**) Venn analysis of the highly susceptible (healthy) and moderate-susceptible (healthy) group.

Subsequently, a comprehensive quantitative gene expression analysis was conducted across these samples. The results revealed that the most significant gene expression differences were found between the highly susceptible (infected) group and the highly resistant group (S_S-vs-R), with a total of 1391 genes showing differential expression. In contrast, the number of differentially expressed genes (DEGs) between the moderate-susceptible (infected) group and the moderate-susceptible (healthy) group (M_S-vs.-M_H) was the lowest, with only 283 genes (Figure 1A). As expected, the total number of differentially expressed genes in the moderate-susceptible (infected) group compared to the highly resistant group (M_S-vs.-R) was less than that in the group (S_S-vs.-R). Further analysis revealed that a large number of DEGs existed in groups based on health status. For example, a large number of DEGs were observed in the comparisons of the highly susceptible (healthy) versus highly resistant (S_H-vs.-R), moderate-susceptible (healthy) versus highly resistant (M_H-vs.-R), and highly susceptible (healthy) versus moderate-susceptible (healthy) groups (S_H-vs.-M_H). Interestingly, there were more upregulated genes than the number of downregulated genes in the infected materials. This pattern was observed in many comparison groups, such as S_S-vs.-R, M_S-vs.-R, S_S-vs.-S_H, and M_S-vs.-M_H. These findings indicate that upregulated expression of genes may be the primary defense mechanism in sugarcane’s reaction to PB diseases.

To examine the various groups of genes that were expressed differently and their intersections, we categorized the DEGs into four groups: highly susceptible group (S) (Figure 1B), moderate-susceptible group (M) (Figure 1C), highly susceptible group (infected) and moderate-susceptible group (infected) (S_S-M_S) (Figure 1D), and highly susceptible group (healthy) and moderate- susceptible group (healthy) (S_H-M_H) (Figure 1E). These groups were divided into seven subsets, denoted by the labels S (1–7), M (1–7), SS_MS (1–7), and SH_MH (1–7).

### 3.2. Functional Annotation of DEGs

**Figure 2:**
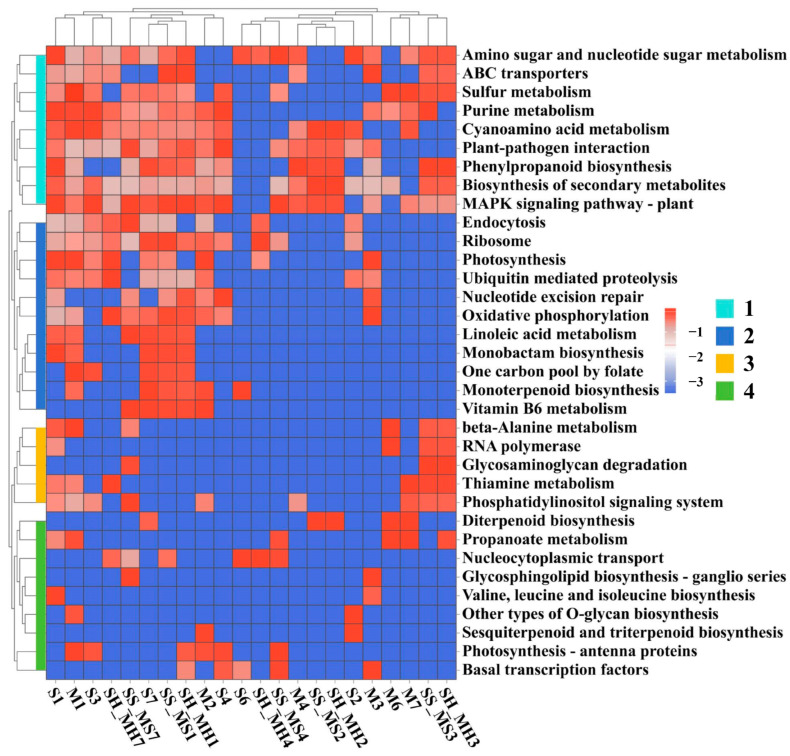
KEGG pathway enrichment analysis on each component of differentially expressed genes. The *p*-value of enrichment to this pathway was treated with −log2 (data+1). The groups S1, S2, S3, S4, S6, S7 represent “Highly Susceptible (Healthy) Materials”, SH_MH1, SH_MH2, SH_MH3, SH_MH4, SH_MH7 represent “Highly Susceptible (Infected) Materials”, M1, M2, M3, M4, M6, M7 represent “Moderate-Susceptible (Healthy) Materials”, and SS_MS1, SS_MS2, SS_MS3, SS_MS4, SS_MS7 represent “Moderate-Susceptible (Infected) Materials”.

To understand the biological functions of the DEGs across the subgroups, a KEGG pathway enrichment analysis was conducted for these genes. The results showed that the DEGs between healthy and infected groups, including the S group (1, 4, 7), the M group (1, 4, 7), the SS_MS group (1, 2, 3), and the SH_MH group (1, 2, 3), were significantly enriched in the plant MAPK signaling pathway (Figure 2). This suggests that the MAPK signaling pathway becomes more active in sugarcane challenged by Pokkah Boeng pathogens, potentially conferring a critical role in the sugarcane’s defense mechanisms against the Pokkah Boeng disease.

The photosynthetic pathway was the most significant enrichment in the S1 and M1 groups, which had the highest number of DEGs. Furthermore, among groups with the same material status, such as the S3, M3, and SH_MH7 groups in the healthy condition, DEGs were mostly found to be enriched in ABC transporters, amino sugar and nucleotide sugar metabolism, and beta-alanine metabolism. In contrast, in the infected state, the DEGs in the SS_MS7 group were enriched in the biological pathways of diterpenoid biosynthesis, glycosaminoglycan degradation, glycosphingolipid biosynthesis—ganglio-series, linoleic acid metabolism, phenylpropanoid biosynthesis, and other biological pathways.

These results of KEGG enrichment analysis indicated that the DEGs were mainly enriched within metabolic and signaling pathways (Group 1), including sugar metabolism, amino acid transport, and plant response mechanisms to pathogens, which are directly or indirectly involved in plant responses to pathogens such as Pokkah Boeng. They play roles in immune responses, energy metabolism, signaling, and maintenance of cellular structures in plants. These functions may reflect different biological strategies such as metabolic adjustments, signal transduction, regulation of gene expression, and secondary metabolite synthesis in response to pathogen attacks. In addition, terpenoids, as important secondary metabolites in plants, play important roles in promoting plant growth, insect resistance, and stress tolerance and may be involved in influencing sugarcane resistance to Pokkah Boeng pathogens. These findings provide new evidence to reveal the resistance mechanism of sugarcane to Pokkah Boeng and offer potential targets for further gene function research and breeding new varieties.

The GO enrichment analysis of DEGs in each fraction revealed that the genes in the S (1, 4) and M (1, 4) groups were significantly enriched for the biological process of response to stimulus (Table 1). The DEGs in the S3 and M3 groups were enriched for biological processes related to posttranscriptional regulation, including translation reinitiation, nuclear-transcribed mRNA catabolic process, and nonsense-mediated decay. These processes are critical for maintaining intracellular protein synthesis homeostasis and gene expression regulation. The DEGs in the SS_MS7 group were mainly enriched in shoot system morphogenesis and leaf morphogenesis.

To explore DEGs between healthy and infected materials, we mined deeper into genes enriched in the plant MAPK signaling pathway, as well as genes enriched in biological processes that respond to stimulus. A total of 17 genes were identified to be enriched in the plant MAPK signaling pathway by removing alleles of the same gene. Among them, 11 genes were from *S. officinarum* and 6 genes were from *S. spontaneum.* The Orthofinder software (v2.5.4) analysis revealed that three genes from *S. officinarum* exhibited homology to four genes from *S. spontaneum*. Specifically, *soff.09G0002280-1C* with *Sspon.03G0008230-3C*; *Soff.00034750* with *Sspon.07G0002940-4D*, *Sspon.02G0018840-4D*; *Soff.00041170* with *Sspon.07G0030050-1C*. NCBI Blast annotation revealed that these genes mostly functioned in response to adversity, signaling, etc. (Table S6). Among them, five genes encode chitinase, an enzyme targeting chitin, a structural component of pathogens. Three genes encode mitogen-activated protein kinase kinase kinase, which is a key component of the plant MAPK signaling pathway, and two genes encode WRKY transcription factors. Notably, three genes are involved in ethylene signaling, indicating that the ethylene signaling pathway and the MAPK signaling pathway may play a synergistic role in sugarcane resistance to Pokkah Boeng diseases.

To identify the key genes, we focused on the intersecting genes enriched in the biological process in both the S1 and M1 groups, and in both the S4 and M4 groups. After eliminating alleles of the same gene, the S (1, 4) and M (1, 4) groups were enriched in a total of 133 genes for the biological process of responding to a stimulus, of which 66 were from *S. officinarum* and 67 were from *S. spontaneum*. By homology analysis, 26 genes in *S. officinarum* were found to be homologous to 36 genes in *S. spontaneum*. These genes enriched in the plant MAPK signaling pathway are also largely involved in influencing plant stress tolerance processes, which is consistent with the genes enriched in the biological process of reacting to stimulus (Table S7). The *Sspon.02G0022580-3C* gene encodes the plant immune receptor protein *RPM1*, which is activated by sensing phosphorylation of RIN4 (*RPM1*-interacting protein 4) at the cell membrane and transmits immune signals downstream to elicit defenses, such as the plant hypersensitive response. Two genes encode RGA2 (Rho GTPases activating protein2) proteins, and one gene encodes a PIK6 protein, which belongs to the plant NBS-LRR gene family and is mainly responsible for activating the defense system to protect the plant.

### 3.3. WGCNA Based on DEGs

**Figure 3:**
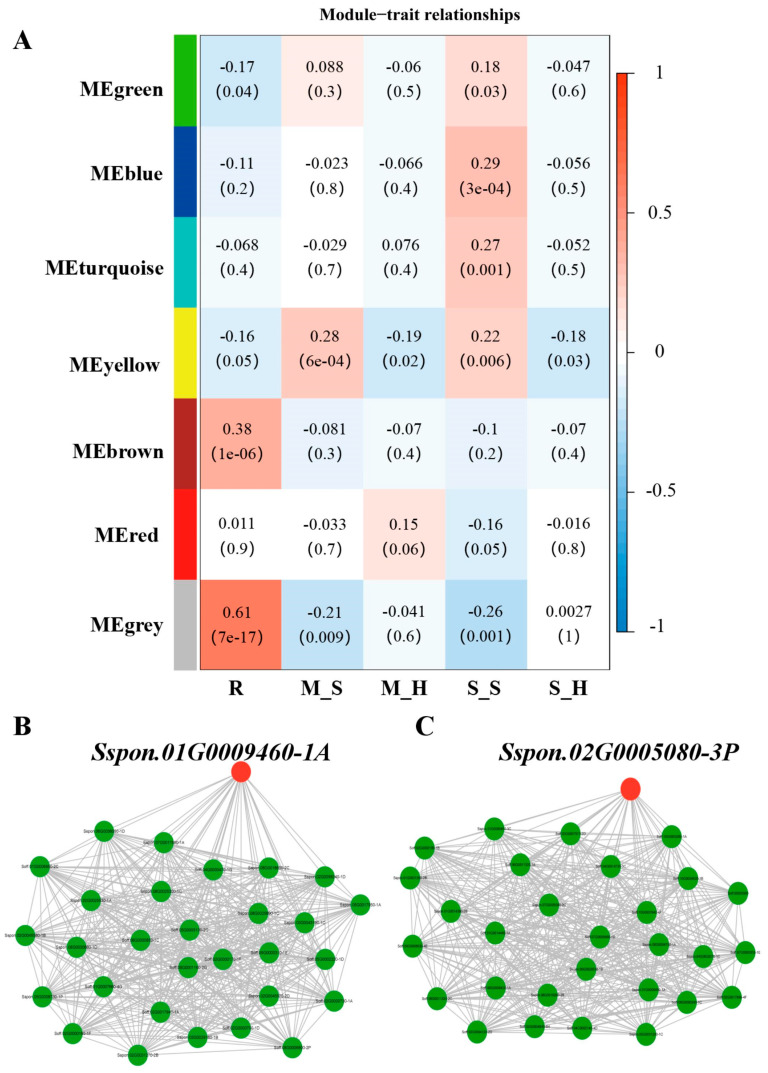
Weighted correlation network analysis of DEGs. (**A**) Gene modules associated with phenotypic traits; (**B**) Cytoscape network visualization of R_MEbrown module; (**C**) Cytoscape network visualization of S_S_MEblue module.

Based on the DEGs obtained from transcriptome analysis, a weighted gene co-expression network analysis was performed using the WGCNA package in R. Screening criteria used the top 75% of the Median absolute deviation (MAD) and a threshold of greater than 0.01. A total of 2186 genes were divided into seven modules associated with phenotypic traits, and correlation heatmaps were generated (Figure 3A). The brown module was highly significant and positively correlated with the highly resistant trait (R), with a correlation coefficient of 0.38 (*p* < 0.01). The yellow module was highly significant and positively correlated with the moderate-susceptible (infected) trait (M_S) and the moderate-susceptible (infected) (M_S) trait (*p* < 0.01), while it was significantly negatively correlated (*p* < 0.05) with both moderate-susceptible (healthy) (M_H) and highly susceptible (healthy) (S_H). In addition, both the turquoise module and the blue module showed a highly significant positive correlation (*p* < 0.01) with the highly susceptible (infected) trait (S_S). In contrast, the green module showed a significant negative correlation (*p* < 0.05) with the highly resistant trait (R) in addition to a significant positive correlation (*p* < 0.05) with the highly susceptible (infected) trait (S_S).

Genes in the module that were significantly associated with the phenotypic traits were further mined. The absolute value of the gene significance (GS) and the correlation value of module membership (MM) were used as the basis for filtering the Hub genes in the module (GS > 0.1 and MM > 0.6). Among them, there were 11 in the R-green module; 65 in the R-brown module; 25 in the M_S-yellow module; 26 in the M_H-yellow module; the highest number of Hub genes (172) in the S_S-turquoise module; 25, 14, and 72 in the S_S-yellow/green/blue modules, respectively, and 15 in the S_H-yellow module. The key gene in the R_brown module was *Sspon.01G0009460-1A* (Figure 3B), and the key gene in the S_S-blue module was *Sspon.02G0005080-3P* (Figure 3C).

In order to clarify the function of Hub genes, these genes were analyzed by KEGG and GO enrichment analyses. The KEGG enrichment results showed that the Hub genes in the R-green module, which was significantly negatively correlated with highly resistant traits, were mainly enriched in the nitrogen metabolism pathway (Table S8). The Hub genes in the modules that were significantly positively associated with M_S-yellow and S_S-yellow were enriched to the ABC transporter protein synthesis pathway. Interestingly, during the analysis of the enrichment of DEGs in the transcriptome of the resistant/susceptible population, it was observed that some of the genes that showed differential expression between the healthy and infected materials were also enriched in the biological pathway mediating the synthesis of ABC transporter proteins.

Hub genes in two other modules, SS-blue and SS-turquoise, which were significantly and positively correlated with the highly susceptible (infected) traits, were enriched to the plant MAPK signaling pathway, which also appeared in the results of the enrichment analysis of the DEGs between healthy and infected materials. The MH-yellow module, which was significantly negatively correlated with the moderate-susceptible (healthy) trait, had Hub genes enriched to the phenylpropanoid biosynthesis pathway. On the other hand, the SH-yellow module, which exhibited a significant negative correlation with the highly susceptible (healthy) trait, had Hub genes that were mainly enriched in the biological pathway of glycosaminoglycan degradation and ABC transporters.

The results of GO enrichment analysis showed (Table S9) that the Hub genes in the R-brown module, which was significantly positively correlated with highly resistant materials, were mainly enriched in defense response, response to stress, response to stimulus, and nitrate transport. In contrast, the R-green module, which was significantly negatively correlated with highly resistant materials, had Hub genes mainly enriched in cellular responses to nitrate, cellular response to reactive nitrogen species, tetrahydrofolate metabolism process, and folic acid-containing compound metabolic process. The module with the most significant correlation with highly susceptible (infected) traits, i.e., SS-blue, had Hub genes mainly enriched in the signaling pathway activated by the auxin-activated signaling pathway, cellular response to auxin stimulus, and oxaloacetate transport.

### 3.4. Identification and Functional Enrichment of Key Genes

By integration of sugarcane NBS-LRR genes identified in the previous study [23], DEGs were obtained from transcriptome analysis of resistant and susceptible materials, and Hub_gene was obtained from WGCNA analysis (Figure S1A). To further explore the functions of the intersected genes, we performed an enrichment analysis of the intersected genes between the components. The KEGG enrichment analysis showed that the intersected genes between DEGs and NBS-LRR were only enriched to the biological pathways of plant–pathogen interaction. The pathways enriched by the intersection of DEGs and WGCNA_hub genes mainly include the plant MAPK signaling pathway, ABC transporter, and galactose metabolism (Table 2).

The results of GO enrichment analysis show that the biological pathways with the most significant and abundant DEGs and NBS-LRR intersection genes are responses to defense, stress, and stimulus (Figure S1B). In contrast, the biological processes with the most abundant DEGs and WGCNA_Hub intersection genes are seed germination, response to stimulus, and response to chemicals; the pathway with the most abundant genes is response to stimulus (Figure S1C).

Genes intersecting across WGCNA_hub, NBS-LRR, and DEGs were prioritized as the most critical for further investigation. NCBI Blast annotation revealed seven genes associated with plant disease resistance (Table 3). For example, the genes *Sspon.06G0027170-1B* and *Sspon.02G0045570-2D* both encode the RGA4 (Rho-type GTPase-activating protein-4) protein, which exhibits resistance to rice blast; the RPP13-like protein 3 encoded by the *Sspon.05G0015970-3D* and *Soff.04G0004430-2B* genes is involved in the plant’s resistance to downy mildew.

### 3.5. RT-qPCR to Validate Candidate Genes

**Figure 4:**
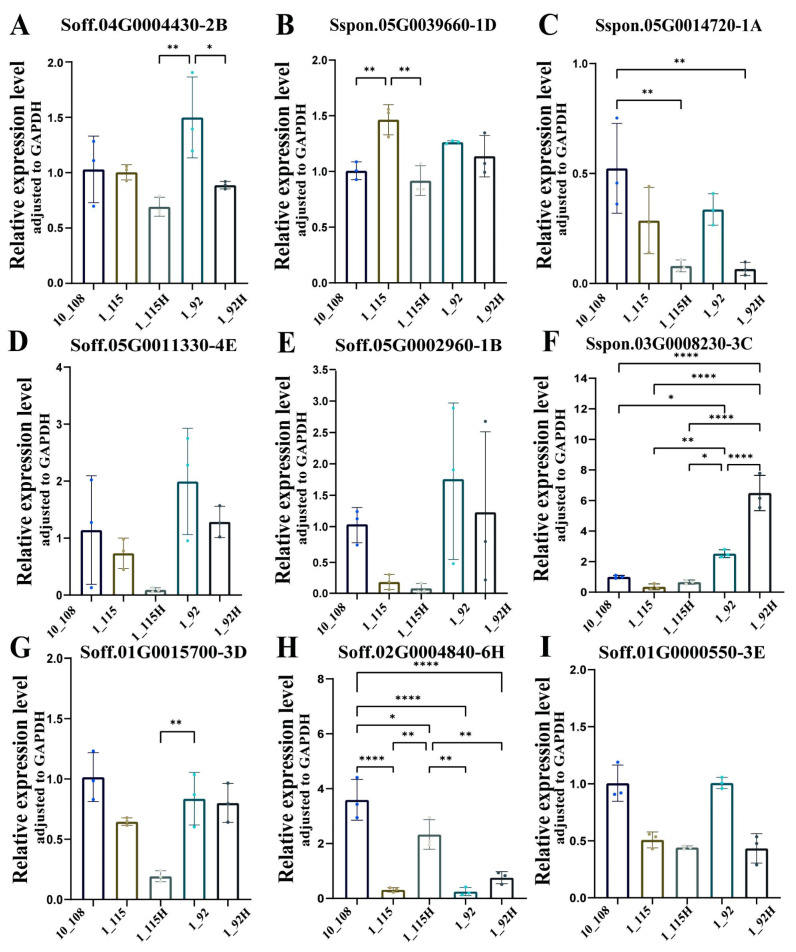
Identification of key genes and RT-qPCR analysis. (**A**–**I**) The RT-qPCR analysis of key regulatory disease resistance genes. * *p* ≤ 0.05; ** *p* ≤ 0.01; **** *p* ≤ 0.0001. (**A**) *Soff.04G0004430-2B*, (**B**) *Sspon.05G0039660-1D*, (**C**) *Sspon.05G0014720-1A*, (**D**) *Soff.05G0011330-4E*, (**E**) *Soff.05G0002960-1B*, (**F**) *Sspon.03G0008230-3C*, (**G**) *Soff.01G0015700-3D*, (**H**) *Soff.02G0004840-6H*, (**I**) *Soff.01G0000550-3E*.

Based on the results of the above analysis, a total of nine candidate genes were selected for real-time fluorescence quantitative PCR analysis in five samples (Highly resistant: 10_108; Moderate-susceptible: 1_115/115H; Highly susceptible: 1_92/92H). These genes include one gene in the intersection of WGCNA_Hub and NBS-LRR (*Soff.05G0011330-4E*); WGCNA_ Hub and NBS-LRR and DEGs intersection of two genes (*Soff.05G0002960-1B*, *Soff.04G0004430-2B*); DEGs and NBS-LRR intersection of two genes (*Sspon.05G0014720-1A*, *Sspon.05G0039660-1D*); WGCNA_ Hub and DEGs intersection of four genes (*Sspon.03G0008230-3C*, *Soff.01G0015700-3D*, *Soff.02G0004840-6H*, *Soff.01G0000550-3E*). There were five NBS-LRR genes and four non-NBS-LRR genes (Figure 4).

In comparing the results of RT-qPCR with those of transcriptome analysis, we observed that the expression trends of all genes were consistent in infected and healthy materials (1_115/115H, and 1_92/92H). However, the patterns of four genes (*Soff.05G0011330-4E*, *Soff.05G0002960-1B*, *Sspon.03G0008230-3C*, and *Soff.01G0015700-3D*) in the highly resistant material (10_108) differed slightly with the transcriptome, which may be due to the differences of the two technologies.

Further analysis revealed that both *Sspon.03G0008230-3C* and *Soff.02G0004840-6H* genes were highly expressed in the healthy materials, whereas all the other genes were highly expressed in the infected materials. Specifically, there was a statistically significant disparity in the expression of the *Sspon.03G0008230-3C* gene in sample 1_92 compared to the other samples (*p* < 0.0001). Additionally, the expression of the *Soff.02G0004840-6H* gene in sample 10_108 compared to sample 1_115, as well as between samples 1_92 and 1_92H, were all extremely significant (*p* < 0.0001). Both *Sspon.03G0008230-3C* and *Soff.02G0004840-6H* genes did not belong to the NBS-LRR gene family. The *Sspon.03G0008230-3C* gene encodes for MAPK kinase kinase (MAPKKK), while the *Soff.02G0004840-6H* gene encodes for a protein with the A20 or AN1 structural domain, specifically the Stress Associated Protein (SAP).

### 3.6. InDel Marker Development and Validation

By utilizing the whole genome resequencing data of the two parental lines and the current transcriptome sequencing data, we screened for variant sites to develop molecular markers that can distinguish between parental materials and highly resistant/susceptible materials. Using the GATK tool for variant detection, a total of 25,598,155 variant sites were detected between the parental lines, including 21,671,034 single nucleotide polymorphisms (SNPs) and 4,107,055 insertion–deletion variants (InDels). A total of 88,664 variant sites, including 70,350 SNPs and 18,314 InDels, were detected in the transcriptome data between the highly resistant/susceptible materials.

This study focused specifically on the InDels linked to the NBS-LRR genes, as these genes have been proposed to have an important role in plant disease resistance. A total of 49,882 InDels were associated with the NBS-LRR genes among the parents, while 323 InDels were associated with the NBS-LRR genes among the highly resistant/susceptible materials. By integrating the InDels obtained from the two sequencing data, 32 InDels were screened out. Two InDels-associated genes identified are part of the intersection between the NBS-LRR genes and Differentially Expressed Genes (DEGs). Specifically, the gene *Sspon.07G0015590_3C* encodes the RGA2 protein, while the gene *Sspon.06G0016070-2B* encodes the Pik-2 protein. These genes are critical components of the plant’s immune response and are likely involved in regulating defense mechanisms against pathogens.

## 4. Discussion

Pokkah Boeng is a critical threat to the sugarcane industry, necessitating effective control strategies to safeguard both yield and quality. Traditional management methods, such as chemical applications, are not only costly and environmentally detrimental but also prone to inducing resistance. Therefore, integrating molecular breeding to develop Pokkah Boeng-resistant sugarcane cultivars can minimize the dependence on chemical pesticides, promoting sustainable sugarcane production. Our study involved a comprehensive transcriptome analysis to explore the genetic segregation in sugarcane associated with resistance or susceptibility to PB. We identified key regulatory and resistance genes by utilizing a combination of differential gene expression analysis and WGCNA incorporating phenotypic features. Notably, our focus on the NBS-LRR gene family provided novel insights into the disease resistance mechanisms of sugarcane Pokkah Boeng. We utilized parental genome resequencing data and transcriptome data from both resistant and susceptible progeny populations to develop InDel markers for molecular marker-assisted breeding.

Through a comparison analysis between healthy and infected material, we identified 17 DEGs. These genes are particularly abundant in the plant MAPK signaling pathway, with three of them encoding MAPK3, a kinase crucial to plant immunological responses. The MAPK pathway, a conserved feature in eukaryotes, consists of three modules of kinases: MAPK kinase kinase (MKKK or MEKK), MAPK kinase (MKK or MEK), and MAPK. This pattern ensures the sequential amplification and transmission of signals to the nucleus, resulting in specific biological responses [24,25]. Research has delineated a more comprehensive MAPK signaling cascade in plant immunity, specifically the MAPKKK3/MAPKKK5-MKK4/MKK5-MPK3/MPK6 pathway, MAPK3 functions upstream of the pathway, and its disruption leads to a reduction in the activity of MPK3/MPK6 that responds to pathogen-associated molecular patterns (PAMPs) and increased susceptibility to pathogens [26]. The initiation of MAPK signaling is among the earliest responses to pathogen detection, with various pattern-recognition receptors (PRRs) on cell membranes known to activate the MAPK signaling pathway upon perception of PAMPs. Notable examples include the bacterial flagellin-sensing 2 (FLS2) and the chitin receptor CERK1 from *Arabidopsis* [27,28]. In this study, differential gene expression analysis revealed significant enrichment in genes associated with the plant MAPK signaling pathway, including six genes encoding chitinases. Chitinases are pathogenesis-related proteins (PR) that contribute to plant immune responses to fungal pathogens. During the process of fungal invasion, the mycelium initially enters the interstitial space of the cell, and the apoplastic chitinases hydrolyze the chitin in the cell wall components of the fungus and release chitin fragments. These fragments bind to their corresponding chitin receptor, are transmitted through the MAPK signaling pathway, and then activate the downstream immune response [29].

Currently, researchers have identified several MAPK kinases and their cascade pathways from plants, such as MEKK1-MKK1/MKK2-MPK4 and MAPKKK3/MAPKKK5-MKK4/MKK5-MPK3/MPK6 cascades in Arabidopsis [26,30]; and *OsMPK2*, *OsMPK4*, *OsMPK5*, *OsMPK7*, *OsMPK8*, *OsMPK12*, *OsMPK13*, *OsMPK15*, and *OsMPK17* respond to rice blast fungus [29,31,32,33]. There is limited research on the involvement of the MAPK signaling pathway in disease resistance in sugarcane. Wu et al. [34] identified three upregulated genes (*ScBAK1*, *ScMapkk*, and *ScGloI*) in smut-infected sugarcane using Solexa sequencing and RT-PCR. The study highlighted *ScMapkk*’s critical role in sugarcane smut resistance. Zhang et al. [35] characterized the *FvBCK1* gene, encoding a MAPKKK homolog, which is essential for various biological processes, including growth, production of microscopic and macroscopic conidia, cell wall integrity, and also for virulence and response to oxidative stress induced by the sugarcane Pokkah Boeng pathogen. In this study, transcriptome profiling of DEGs between healthy and Pokkah Boeng-infected sugarcane revealed significant enrichment in the plant MAPK signaling pathway. The WGCNA analysis confirmed this finding, demonstrating that hub genes linked to highly susceptible (infected) modules were significantly enriched in the MAPK pathway. Our findings indicate that the MAPK signaling system is activated by Pokkah Boeng stress, playing a pivotal role in modulating downstream immune responses in sugarcane. Activation of this pathway can initiate a sequence of defense mechanisms, including the synthesis of disease-associated proteins, activation of immune-related gene expression, and enhancement of cell wall defenses. Additionally, the MAPK pathway may interact with other signaling pathways, contributing to a complex network that regulates sugarcane’s immune response.

In this study, WGCNA revealed gene networks linked to sugarcane Pokkah Boeng resistance, with certain modules showing significant trait correlations. The brown module’s significant positive connection with the highly resistant trait (R) indicates that it plays a crucial role in disease defense. Furthermore, the yellow module exhibits positive correlations with the moderate-susceptible (infected) (M_S) and highly susceptible (infected) (S_S) phenotypes while displaying negative correlations with healthy phenotypes. This emphasizes the significant contribution of genes within these modules to the progression of the disease. We identified key hub genes as potential targets for further research by correlating gene expression with module eigengenes and phenotype traits. KEGG enrichment analysis showed that Hub genes in the R-green module, which was significantly negatively correlated with the highly resistant trait, were predominantly enriched in the nitrogen metabolism pathway. This indicates nitrogen metabolism plays a regulatory role in disease resistance. The enrichment of Hub genes in the modules SS-blue and SS-turquoise, which were positively correlated with highly susceptible (infected) traits, revealed the importance of the ABC transporter protein synthesis pathway and the plant MAPK signaling pathway in response to disease. ABC transporter proteins are involved in several key processes, which may be involved in the plant’s initial recognition of and response to pathogens by regulating the cell membrane molecular composition to enhance plant defense barriers [36]. Through these functions, ABC transporter proteins may contribute to strengthening the immune response of plants. A comprehensive analysis of the interactions and regulatory networks among these genes will help to reveal the complexity of sugarcane’s response to Pokkah Boeng.

This study’s intersection of WGCNA_hub and NBS-LRR and DEGs yielded two common genes: *Sspon.06G0027170-1B* and *Sspon.02G0045570-2D*, both of which are NBS-LRR genes encoding RGA4 proteins. Studies using rice protoplasts have shown that RGA4 triggers a cell death response independent of pathogen-derived avirulence (AVR) proteins. This response is normally inhibited by RGA5, a Rho-type GTPase-activating protein. However, when the pathogen effector AVR-Pia is directly recognized and binds to RGA5, the inhibition is removed, resulting in cellular death. This mechanism serves as a defense mechanism against pathogens [37,38]. Interestingly, the gene *Soff.05G0007900-1A*, encoding the RGA5 protein, was identified in association with the key gene for RGA4, which is linked to our molecular marker for distinguished sugarcane’s resistance to Pokkah Boeng. This finding suggests that both RGA4 and RGA5 proteins are likely involved in sugarcane resistance to Pokkah Boeng and may have a significant impact. The RGA2 protein encoded by the intersecting gene *Sspon.06G0036050-1D* gene is involved in resistance to *Fusarium oxysporum* f. sp. *cubense* tropical race 4 (TR4) in banana [39]. Additionally, this protein is also encoded by another gene associated with our molecular markers, *Sspon.07G0015590-3C*, which further highlights the significance of the RGA2 protein family in plant defense against fungal diseases. This further highlights the significance of the RGA2 protein family in enhancing plant resistance against various fungal diseases. Two other intersecting genes, *Sspon.05G0015970-3D* and *Soff.04G0004430-2B*, both encode RPP13-like protein 3, and it has been shown that RPP13-like protein 3 exhibits downy mildew pathogen (*Peronospora parasitica*) resistance [40]. For PIK6-NP-like encoded by the *Soff.05G0002960-1B* gene, there is no clarity on its function, but a study confirmed that the PIK protein exhibits resistance to rice blast [41]. Our research indicates that RGA4/5, RPP13-like protein 3, and RGA2, which provide resistance against rice blast, downy mildew, and TR4, respectively, exhibit a shared characteristic in their ability to resist fungal diseases, even if they target different species that cause these illnesses. We hypothesize that similar to the multispecies resistance genes mentioned in the above study, there is also a subset of resistance genes that are broad-spectrum against fungal diseases in plants. These genes may have been naturally selected and preserved during the prolonged evolutionary process of plants.

Furthermore, the RT-qPCR analysis revealed significant differences in the expression levels of two DEGs and the intersection genes of WGCNA_Hub_gene (*Sspon.03G0008230-3C* and *Soff.02G0004840-6H*) across the samples. Notably, both of these genes exhibited higher expression levels in healthy materials. The gene function search identified that the *Sspon.03G0008230-3C* gene encodes a MAPK kinase kinase, which serves as the first cascade of the MAPK signaling pathway to receive and transmit immune signals. On the other hand, the *Sspon.02G0004840-6H* gene encodes a protein with the structural domains of A20/AN1, specifically the Stress Associated Protein (SAP) [42,43]. It is worth mentioning that SAP proteins may play an important role in immune regulation in sugarcane, although their study in plants has mainly focused on the response to abiotic stresses. Tyagi et al. [44] demonstrated that transgenic tobacco overexpressing *OsSAP1* had enhanced resistance to *Pseudomonas syringae* and Gao et al. [45] found that cotton with silenced *GhSAP17A/D* exhibited improved resistance to *Verticillium dahliae*. These findings indicate a key role for SAP proteins in plant immunity. The *Soff.02G0004840 -6H* genes exhibited significant variations in expression levels among the healthy and infected materials in the moderate-susceptible group, the healthy and infected materials in the highly susceptible group, and the susceptible and resistant materials. Notably, the differences in expression between the resistant and susceptible materials were particularly significant. It is hypothesized that SAP proteins likely have a significant function in sugarcane’s defense against Pokkah Boeng disease. However, additional investigations are required to clarify their precise defense strategies.

Molecular markers play a crucial role in genomics and molecular biology, especially for practical purposes in breeding. Beyond traditional resequencing for variant detection, researchers are increasingly using transcriptome sequencing to create molecular markers. Meng et al. [46] utilized RNA-seq and ARMS-PCR to develop SNP markers in sweet potato. Similarly, Muñoz-Espinoza [47] identified a set of informative and transferable SNP and InDel markers associated with berry size using RNA-seq. This approach rapidly captures functional gene transcriptional information without genome-wide data requirements, linking genetic information directly to biological functions and enhancing the efficiency of molecular breeding. In this study, we integrated parental resequencing and progeny transcriptome data to develop 32 InDels. We have identified two InDels-associated NBS-LRR genes that exhibit differential expression patterns in response to pathogenic stress. These genes have been reported in the plant’s immune system. The elucidation of these genes enhances our comprehension of the genetic framework of disease resistance in sugarcane and may offer novel insights into the development of resistance strategies against various pathogens. These markers are promising for future molecular-assisted breeding of disease-resistant sugarcane.

## 5. Conclusions

Our study utilized transcriptome sequencing and bioinformatics to identify key genetic factors and molecular markers associated with sugarcane resistance to Pokkah Boeng disease. We uncovered differentially expressed genes and gene modules linked to Pokkah Boeng resistance, particularly in the MAPK signaling and ABC transporter pathways. We developed a series of putative molecular markers that potentially efficiently distinguish between highly resistant and susceptible materials. This research offers critical genetic resources and innovative molecular tools essential for enhancing sugarcane’s defense against Pokkah Boeng resistance.

## Figures and Tables

**Table 1:** GO enrichment analysis of DEGs in different groups of sugarcane.

Type of Material	Group	Biological Pathway
Healthy materials-vs.-Sick materials	S (1,4) and M (1,4)	Response to stimulus
Healthy materials-vs.-Healthy materials	S 3 and M 3	Translation reinitiation Nuclear-transcribed mRNA catabolic process Nonsense-mediated decay
Sick materials-vs.-Sick materials	SS_MS7	Shoot system morphogenesis Leaf morphogenesis Simple leaf morphogenesis

**Table 2:** KEGG enrichment analysis of key genes.

Group	Biological Pathway
DEGs and NBS-LRR	Plant–pathogen interaction
DEGs and WGCNA_Hub	MAPK signaling pathway–plant
	ABC transporters
	Galactose metabolism

**Table 3:** WGCNA_hub and NBS-LRR and DEGs intersection gene function finding.

Gene_Name	Gene/Protein	Function Description
*Sspon.05G0015970-3D*	RPP13-like protein 3	Resistance to downy mildew
*Sspon.06G0027170-1B*	RGA4	Disease resistance (rice blast)
*Sspon.06G0036050-1D*	RGA2	Disease resistance
*Sspon.02G0045570-2D*	RGA4	Disease resistance (rice blast)
*Sspon.08G0005030-1A*	None	None
*Soff.05G0002960-1B*	PIK6-NP-like	Disease resistance
*Soff.04G0004430-2B*	RPP13-like protein 3	Resistance to downy mildew
*Soff.00065450*	RPS2	Resistance to *P. syringae*

## Data Availability

The sequencing data have been deposited in the BIG Submission Portal (BIG Sub), managed by the China National Center for Bioinformation (CNCB), under the accession number CRA019156. https://ngdc.cncb.ac.cn/gsa/s/E87cPVa5 (accessed on 28 November 2024).

## Supplementary Materials

The following supporting information can be downloaded at: https://www.mdpi.com/article/10.3390/plants13243497/s1, Figure S1: Identification and functional enrichment of key regulatory and disease resistance genes; Table S1: Standard for classification of Sugarcane Pokkah boeng disease; Table S2: The Phenology survey of sugarcane Pokkah boeng disease; Table S3: RT-qPCR primer sequence; Table S4: Molecular marker primer sequences; Table S5: The results of transcriptome data quality control; Table S6: Enrichment of genes in the MAPK signaling pathway in plant; Table S7: The DEGs enriched in biological processes in response to stimulus; Table S8: The KEGG enrichment analysis of Hub genes; Table S9: The GO enrichment analysis of Hub genes.

## Abbreviations

PB	Pokkah Boeng
DEGs	Differentially expressed genes
SSR	Simple sequence repeat
WGCNA	Weighted gene co-expression network analysis
NBS-LRR	Nucleotide-binding sites and leucine-rich repeat
TPM	Transcripts per million
FDR	False discovery rate
MAD	Median absolute deviation
RGA2	Rho GTPases activating protein2
MAPK	Mitogen-activated protein kinase
GS	Gene significance
MM	Module membership
RGA4	Rho-type GTPase-activating protein-4
SAP	Stress associated protein
SNPs	Single nucleotide polymorphisms
InDels	Insertion–Deletion variants
PAMPs	Pathogen-associated molecular patterns
FLS2	Flagellin-sensing 2
PR	Pathogenesis-related proteins
AVR	Avirulence
