# Supplementary material for: Mining of Candidate Genes and Developing Molecular Markers Associated with Pokkah Boeng Resistance in Sugarcane (Saccharum spp.)
Source: Plants (Basel). 2024 Dec 14;13(24):3497. doi: 10.3390/plants13243497 (PMC11678196; doi:10.3390/plants13243497)
Supplement: Supplementary file 1 [file plants-13-03497-s001.zip › plants-3315440-supplementary.pdf]

Table S1 Standard for classification of Sugarcane Pokkah boeng disease

Morbidity level	Onset symptoms
0	No symptoms of Pokkah boeng
1	1~2 leaves are infected, the base or other parts of the leaves are chlorosis, slightly wrinkled, and the spindle was slightly wrinkled, with a few reddish stripes and spots appearing in the chlorotic part.
2	3 leaves infected, the Heart leaf were wrinkled in the shape of a ladder, significantly yellowed, twisted and reddish stripes, twisted or notched leaf twine, and brown spots appear
3	Heart leaf rot, top rot, obvious red stripes and a large number of white sporangia.。
4	Complete rotting of the heart leaves, and death of the whole plant

Table S2 The Phenology survey of sugarcane Pokkah boeng disease

Sample	Repeat 1							Repeat 2							Repeat 3							Average incidence rate （%）	Average condition index （%）
	Tiller numbers	1	2	3	4	incidence rate （%）	condition index （%）	Tiller numbers	1	2	3	4	incidence rate （%）	condition index （%）	Tiller numbers	1	2	3	4	incidence rate（%）	condition index （%）		
10_108	30	0	0	0	0	0.00%	0.00%	23	0	0	0	0	0.00%	0.00%	28	0	0	0	0	0.00%	0.00%	0.00%	0.00%
10_123	46	0	0	0	0	0.00%	0.00%	47	0	0	0	0	0.00%	0.00%	48	0	0	0	0	0.00%	0.00%	0.00%	0.00%
1_102	32	0	0	0	0	0.00%	0.00%	34	0	0	0	0	0.00%	0.00%	22	0	0	0	0	0.00%	0.00%	0.00%	0.00%
1_115	5	0	3	0	0	60.00%	30.00%	42	4	1	0	2	16.67%	8.33%	44	1	1	3	0	11.36%	6.82%	29.34%	15.05%
1_126	18	0	2	4	0	33.33%	22.22%	12	1	2	3	0	50.00%	29.17%	13	2	1	3	0	46.15%	25.00%	43.16%	25.46%
1_29	33	0	1	0	0	3.03%	1.52%	41	0	0	0	0	0.00%	0.00%	31	0	0	0	0	0.00%	0.00%	1.01%	0.51%
14_42	24	0	0	0	0	0.00%	0.00%	0	0	0	0	0	0.00%	0.00%	0	0	0	0	0	0.00%	0.00%	0.00%	0.00%
1_48	23	0	0	0	0	0.00%	0.00%	48	0	0	0	0	0.00%	0.00%	30	0	0	0	0	0.00%	0.00%	0.00%	0.00%
1_55	16	0	1	0	0	6.25%	3.13%	39	0	3	0	0	7.69%	3.85%	21	1	1	2	1	23.81%	15.48%	12.58%	7.48%
1_58	30	0	3	2	0	16.67%	10.00%	6	1	0	1	0	33.33%	16.67%	23	0	0	2	0	8.70%	6.52%	19.57%	11.06%
16_101	17	0	0	0	0	0.00%	0.00%	0	0	0	0	0	0.00%	0.00%	0	0	0	0	0	0.00%	0.00%	0.00%	0.00%
1_77	26	0	2	0	0	7.69%	3.85%	54	0	0	0	0	0.00%	0.00%	45	0	0	0	0	0.00%	0.00%	2.56%	1.28%
1_78	52	21	5	3	4	63.46%	26.92%	43	15	6	4	0	58.14%	22.67%	40	11	2	6	0	47.50%	20.63%	53.4%	23.40%
1_92	10	0	2	0	0	20.00%	10.00%	17	0	2	7	1	58.82%	42.65%	22	1	0	4	0	22.73%	14.77%	33.85%	22.47%
20_81	40	0	0	0	0	0.00%	0.00%	18	0	0	0	0	0.00%	0.00%	11	0	0	0	0	0.00%	0.00%	0.00%	0.00%
2_105	31	0	2	0	0	3.23%	1.61%	43	0	0	0	0	0.00%	0.00%	28	0	0	0	0	0.00%	0.00%	1.08%	0.53%
2_107	27	0	1	0	0	3.70%	1.85%	34	0	0	0	0	0.00%	0.00%	19	0	0	0	0	0.00%	0.00%	1.23%	0.62%
21_141	0	0	0	0	0	0.00%	0.00%	0	0	0	0	0	0.00%	0.00%	0	0	0	0	0	0.00%	0.00%	0.00%	0.00%
21_177	50	0	0	0	0	0.00%	0.00%	27	0	0	0	0	0.00%	0.00%	44	0	0	0	0	0.00%	0.00%	0.00%	0.00%

2_129	35	7	2	1	1	31.43%	12.86%	0	0	0	0	0	0.00%	0.00%	0	0	0	0	0	0.00%	0.00%	10.48%	4.29%
2_14	37	0	1	4	1	16.22%	12.16%	14	0	0	0	0	0.00%	0.00%	37	0	0	0	0	0.00%	0.00%	5.41%	4.05%
22_131	54	0	0	0	0	0.00%	0.00%	40	0	3	0	0	7.50%	3.75%	26	0	0	0	0	0.00%	0.00%	2.50%	1.25%
22_22	0	0	0	0	0	0.00%	0.00%	0	0	0	0	0	0.00%	0.00%	0	0	0	0	0	0.00%	0.00%	0.00%	0.00%
23_109	5	0	2	0	0	40.00%	20.00%	4	0	0	0	0	0.00%	0.00%	27	0	0	0	0	0.00%	0.00%	13.33%	6.67%
23_15	4	0	0	0	0	0.00%	0.00%	26	0	0	0	0	0.00%	0.00%	38	0	0	0	0	0.00%	0.00%	0.00%	0.00%
23_166	50	0	0	0	0	0.00%	0.00%	36	0	0	0	0	0.00%	0.00%	15	0	0	0	0	0.00%	0.00%	0.00%	0.00%
23_194	29	6	1	3	0	34.48%	14.66%	25	0	0	0	0	0.00%	0.00%	15	3	1	7	1	80.00%	50.00%	38.16%	21.55%
23_196	11	3	1	0	0	36.36%	11.36%	0	0	0	0	0	0.00%	0.00%	0	0	0	0	0	0.00%	0.00%	12.12%	3.79%
24_142	28	0	0	0	0	0.00%	0.00%	34	2	2	7	3	41.18%	28.68%	19	2	3	4	3	63.16%	42.11%	34.78%	23.59%
24_148	16	0	0	0	0	0.00%	0.00%	50	0	0	0	0	0.00%	0.00%	29	0	0	0	0	0.00%	0.00%	0.00%	0.00%
24_165	10	0	2	0	1	30.00%	20.00%	11	0	0	1	3	36.36%	34.09%	13	0	0	0	0	0.00%	0.00%	22.12%	18.03%
24_97	45	0	2	0	0	4.44%	2.22%	38	0	0	0	0	0.00%	0.00%	17	0	0	0	0	0.00%	0.00%	1.48%	0.74%
2_50	62	0	0	0	0	0.00%	0.00%	47	0	0	0	0	0.00%	0.00%	15	0	0	0	0	0.00%	0.00%	0.00%	0.00%
2_53	23	0	0	0	0	0.00%	0.00%	8	0	0	0	0	0.00%	0.00%	21	1	1	3	0	23.81%	14.29%	7.94%	4.76%
25_55	9	0	0	0	0	0.00%	0.00%	5	0	0	0	0	0.00%	0.00%	25	0	0	0	0	0.00%	0.00%	0.00%	0.00%
25_56	15	0	0	0	0	0.00%	0.00%	20	0	0	0	0	0.00%	0.00%	19	0	0	0	0	0.00%	0.00%	0.00%	0.00%
25_58	11	0	0	0	0	0.00%	0.00%	19	0	2	0	0	10.53%	5.26%	26	0	0	0	0	0.00%	0.00%	3.51%	1.75%
25_81	20	0	0	0	0	0.00%	0.00%	26	0	0	0	0	0.00%	0.00%	0	0	0	0	0	0.00%	0.00%	0.00%	0.00%
2_60	4	2	0	0	0	50.00%	12.50%	37	0	1	0	0	2.70%	1.35%	16	1	0	3	0	25.00%	15.63%	25.90%	9.83%
26_122	26	1	2	3	0	23.08%	13.46%	3	0	0	0	0	0.00%	0.00%	0	0	0	0	0	0.00%	0.00%	7.69%	4.49%
26_88	41	0	0	0	0	0.00%	0.00%	36	0	0	0	0	0.00%	0.00%	40	0	0	0	0	0.00%	0.00%	0.00%	0.00%
26_97	0	0	0	0	0	0.00%	0.00%	0	0	0	0	0	0.00%	0.00%	0	0	0	0	0	0.00%	0.00%	0.00%	0.00%
27_102	21	1	1	0	0	9.52%	3.57%	32	1	2	0	0	9.38%	3.91%	16	0	0	0	0	0.00%	0.00%	6.30%	2.49%
27_175	1	0	1	0	0	100.00%	50.00%	0	0	0	0	0	0.00%	0.00%	0	0	0	0	0	0.00%	0.00%	33.33%	16.67%
27_23	33	0	0	0	0	0.00%	0.00%	25	0	0	0	0	0.00%	0.00%	34	0	0	0	0	0.00%	0.00%	0.00%	0.00%

27_28	30	1	0	0	0	3.33%	0.83%	44	0	1	1	0	4.55%	2.84%	45	0	0	0	0	0.00%	0.00%	2.63%	1.22%
27_30	22	0	3	0	0	13.64%	6.82%	21	0	0	0	0	0.00%	0.00%	48	0	0	0	0	0.00%	0.00%	4.55%	2.27%
27_44	22	1	0	0	1	9.09%	5.68%	33	0	0	0	0	0.00%	0.00%	10	0	0	0	0	0.00%	0.00%	3.03%	1.89%
27_79	24	0	0	0	0	0.00%	0.00%	36	0	0	0	0	0.00%	0.00%	30	0	0	0	0	0.00%	0.00%	0.00%	0.00%
27_95	32	0	4	4	0	25.00%	15.63%	10	0	1	4	0	50.00%	35.00%	51	3	7	11	7	54.90%	38.24%	43.30%	29.62%
28_105	4	0	0	0	0	0.00%	0.00%	15	0	0	0	0	0.00%	0.00%	8	1	1	1	1	50.00%	31.25%	16.67%	10.42%
28_151	41	0	0	0	0	0.00%	0.00%	79	0	0	0	0	0.00%	0.00%	41	0	0	0	0	0.00%	0.00%	0.00%	0.00%
28_161	17	0	0	0	0	0.00%	0.00%	14	0	0	0	0	0.00%	0.00%	11	0	0	0	0	0.00%	0.00%	0.00%	0.00%
28_21	11	0	2	1	1	36.36%	25.00%	21	0	2	1	0	14.29%	8.33%	16	2	2	0	0	25.00%	9.38%	25.22%	14.24%
28_31	30	6	4	2	1	36.36%	20.00%	51	15	8	4	0	52.94%	21.08%	23	5	1	4	0	43.48%	20.65%	44.26%	20.58%
28_35	25	0	0	0	0	0.00%	0.00%	49	0	0	0	0	0.00%	0.00%	21	0	0	0	0	0.00%	0.00%	0.00%	0.00%
28_6	23	0	0	0	0	0.00%	0.00%	25	0	0	0	0	0.00%	0.00%	23	0	0	0	0	0.00%	0.00%	0.00%	0.00%
28_61	8	0	0	1	0	12.50%	9.38%	29	0	1	3	0	13.79%	9.48%	8	0	0	0	0	0.00%	0.00%	8.76%	6.29%
29_172	0	0	0	0	0	0.00%	0.00%	0	0	0	0	0	0.00%	0.00%	0	0	0	0	0	0.00%	0.00%	0.00%	0.00%
2_95	25	0	0	0	0	0.00%	0.00%	33	0	0	0	0	0.00%	0.00%	53	0	0	0	0	0.00%	0.00%	0.00%	0.00%
30_107	11	0	0	0	0	0.00%	0.00%	33	0	0	0	0	0.00%	0.00%	12	1	1	0	0	16.67%	6.25%	5.56%	2.08%
30_109	14	0	2	0	0	14.29%	7.14%	67	0	0	0	0	0.00%	0.00%	25	0	0	0	0	0.00%	0.00%	4.76%	2.38%
30_146	40	0	0	0	0	0.00%	0.00%	57	0	0	0	0	0.00%	0.00%	36	0	0	0	0	0.00%	0.00%	0.00%	0.00%
30_152	13	0	2	0	0	15.38%	7.69%	7	0	0	0	0	0.00%	0.00%	10	0	0	0	0	0.00%	0.00%	5.13%	2.56%
30_169	23	0	5	2	0	30.43%	17.39%	11	2	2	0	1	45.45%	22.73%	16	3	0	1	2	37.50%	21.88%	37.79%	20.66%
31_110	0	0	0	0	0	0.00%	0.00%	14	0	2	0	0	14.29%	7.14%	0	0	0	0	0	0.00%	0.00%	4.76%	2.38%
3_120	45	8	5	5	1	42.22%	20.56%	23	4	5	3	1	56.52%	29.35%	0	0	0	0	0	0.00%	0.00%	49.37%	24.96%
3_123	15	0	0	0	0	0.00%	0.00%	12	0	0	3	1	33.33%	27.08%	16	0	2	0	0	12.50%	6.25%	15.28%	11.11%
3_126	29	0	0	0	0	0.00%	0.00%	27	0	0	0	0	0.00%	0.00%	0	0	0	0	0	0.00%	0.00%	0.00%	0.00%
3_143	9	0	1	2	1	44.44%	33.33%	40	10	4	4	2	50.00%	23.75%	0	0	0	0	0	0.00%	0.00%	147.22%	28.54%
31_76	26	0	0	0	0	0.00%	0.00%	31	1	2	0	0	9.68%	4.03%	22	0	0	0	0	0.00%	0.00%	3.23%	1.34%

31_78	31	0	0	0	0	0.00%	0.00%	25	0	0	0	0	0.00%	0.00%	10	0	0	0	0	0.00%	0.00%	0.00%	0.00%
34_103	16	0	0	0	0	0.00%	0.00%	42	0	4	0	0	9.52%	4.76%	40	5	2	2	1	25.00%	11.88%	11.51%	5.55%
3_43	34	2	3	0	1	17.65%	8.82%	0	0	0	0	0	0.00%	0.00%	0	0	0	0	0	0.00%	0.00%	5.88%	2.94%
34_71	57	0	1	2	0	5.26%	3.51%	21	0	0	0	0	0.00%	0.00%	39	0	0	0	0	0.00%	0.00%	1.75%	1.17%
34_77	17	0	4	1	1	35.29%	22.06%	52	5	4	6	6	40.38%	26.44%	71	18	5	3	4	42.25%	18.66%	39.31%	22.39%
34_83	23	0	0	0	0	0.00%	0.00%	11	0	0	3	1	36.36%	29.55%	9	1	2	1	0	44.44%	22.22%	26.94%	17.26%
35_148	25	0	3	0	0	12.00%	6.00%	37	0	2	0	0	5.41%	2.70%	25	0	0	0	0	0.00%	0.00%	5.80%	2.90%
36_105	26	2	5	11	2	76.92%	50.96%	23	4	2	4	2	52.17%	30.43%	22	2	1	3	4	45.45%	32.95%	58.18%	38.12%
3_69	4	0	0	0	0	0.00%	0.00%	24	0	5	1	0	25.00%	13.54%	18	0	0	0	0	0.00%	0.00%	8.33%	4.51%
36_95	12	0	0	0	0	0.00%	0.00%	53	0	0	0	0	0.00%	0.00%	12	0	0	0	0	0.00%	0.00%	0.00%	0.00%
3_77	17	0	4	1	1	35.29%	22.06%	52	5	4	6	6	40.38%	26.44%	71	0	5	3	0	11.27%	6.69%	28.98%	18.40%
37_72	1	0	0	0	0	0.00%	0.00%	11	0	0	0	0	0.00%	0.00%	11	0	0	0	0	0.00%	0.00%	0.00%	0.00%
3_81	25	0	0	0	0	0.00%	0.00%	34	0	0	0	0	0.00%	0.00%	17	0	0	0	0	0.00%	0.00%	0.00%	0.00%
38_115	25	0	0	0	0	0.00%	0.00%	22	0	0	0	0	0.00%	0.00%	0	0	0	0	0	0.00%	0.00%	0.00%	0.00%
38_139	39	0	2	0	0	5.13%	2.56%	12	0	0	0	0	0.00%	0.00%	0	0	0	0	0	0.00%	0.00%	1.71%	0.85%
38_155	8	0	0	0	0	0.00%	0.00%	0	0	0	0	0	0.00%	0.00%	0	0	0	0	0	0.00%	0.00%	0.00%	0.00%
38_193	8	0	0	0	0	0.00%	0.00%	0	0	0	0	0	0.00%	0.00%	0	0	0	0	0	0.00%	0.00%	0.00%	0.00%
39_126	18	0	0	0	0	0.00%	0.00%	26	0	0	0	0	0.00%	0.00%	0	0	0	0	0	0.00%	0.00%	0.00%	0.00%
39_136	0	0	0	0	0	0.00%	0.00%	33	0	0	0	0	0.00%	0.00%	30	0	0	0	0	0.00%	0.00%	0.00%	0.00%
39_142	39	1	0	0	0	2.56%	0.64%	0	0	0	0	0	0.00%	0.00%	0	0	0	0	0	0.00%	0.00%	0.85%	0.21%
39_185	75	6	1	0	0	9.33%	2.67%	23	0	0	1	0	4.35%	3.26%	46	0	0	0	0	0.00%	0.00%	4.56%	1.98%
39_198	41	0	0	0	0	0.00%	0.00%	79	0	0	0	0	0.00%	0.00%	50	0	0	0	0	0.00%	0.00%	0.00%	0.00%
3_92	25	0	0	0	0	0.00%	0.00%	30	0	1	0	0	3.33%	1.67%	37	0	0	0	0	0.00%	0.00%	1.11%	0.56%
39_3	28	0	2	4	0	21.43%	14.29%	33	0	0	0	0	0.00%	0.00%	22	1	1	2	0	18.18%	10.23%	13.20%	8.17%
3_93	25	0	1	1	0	8.00%	5.00%	0	0	0	0	0	0.00%	0.00%	18	0	0	0	0	0.00%	0.00%	2.67%	1.67%
39_40	44	0	0	0	0	0.00%	0.00%	38	1	2	0	0	7.89%	3.29%	50	0	0	0	0	0.00%	0.00%	2.63%	1.10%

39_42	4	0	1	0	0	25.00%	12.50%	14	0	0	0	0	0.00%	0.00%	0	0	0	0	0	0.00%	0.00%	8.33%	4.17%
39_73	11	1	0	0	0	9.09%	2.27%	13	0	0	0	0	0.00%	0.00%	12	0	0	0	0	0.00%	0.00%	3.03%	0.76%
40_173	0	0	0	0	0	0.00%	0.00%	6	0	0	0	0	0.00%	0.00%	10	0	0	0	0	0.00%	0.00%	0.00%	0.00%
40_180	5	0	0	0	0	0.00%	0.00%	14	0	0	0	0	0.00%	0.00%	13	0	0	0	0	0.00%	0.00%	0.00%	0.00%
4_111	6	0	3	0	0	50.00%	25.00%	69	0	0	0	0	0.00%	0.00%	55	0	0	0	0	0.00%	0.00%	16.67%	8.33%
4_122	18	0	0	0	0	0.00%	0.00%	6	0	0	0	0	0.00%	0.00%	35	1	3	2	0	17.14%	9.29%	5.71%	3.10%
4_139	18	0	0	0	0	0.00%	0.00%	37	0	4	1	0	13.51%	7.43%	0	0	0	0	0	0.00%	0.00%	4.50%	2.48%
4_157	22	2	0	0	0	9.09%	2.27%	26	0	0	0	0	0.00%	0.00%	14	0	0	0	0	0.00%	0.00%	3.03%	0.76%
4_158	23	0	3	2	1	26.09%	17.39%	0	0	0	0	0	0.00%	0.00%	0	0	0	0	0	0.00%	0.00%	8.70%	5.80%
4_16	16	0	1	2	0	18.75%	12.50%	11	0	0	0	0	0.00%	0.00%	9	0	0	0	0	0.00%	0.00%	6.25%	4.17%
4_166	27	0	3	11	3	62.96%	47.22%	19	0	1	5	0	31.58%	22.37%	29	3	2	3	1	31.03%	17.24%	41.86%	28.94%
4_189	35	0	2	0	2	11.43%	8.57%	45	0	3	2	0	11.11%	6.67%	27	5	2	4	1	44.44%	23.15%	22.33%	12.80%
42_177	0	0	0	0	0	0.00%	0.00%	0	0	0	0	0	0.00%	0.00%	30	0	0	0	0	0.00%	0.00%	0.00%	0.00%
4_28	22	5	0	2	2	40.91%	21.59%	33	6	4	1	3	42.42%	21.97%	0	0	0	0	0	0.00%	0.00%	41.66%	21.78%
43_164	40	0	0	0	0	0.00%	0.00%	45	0	0	0	0	0.00%	0.00%	49	0	0	0	0	0.00%	0.00%	0.00%	0.00%
43_34	18	1	3	3	0	38.89%	22.22%	30	1	1	4	0	20.00%	12.50%	30	2	3	3	0	26.67%	14.17%	28.52%	16.30%
43_51	25	1	0	0	0	4.00%	1.00%	58	1	0	0	0	1.72%	0.43%	19	0	0	0	0	0.00%	0.00%	1.91%	0.48%
4_37	30	0	0	0	0	0.00%	0.00%	42	0	0	0	0	0.00%	0.00%	17	0	0	0	0	0.00%	0.00%	0.00%	0.00%
45_156	11	0	2	0	0	18.18%	9.09%	32	0	3	0	0	9.38%	4.69%	18	1	2	2	0	27.78%	15.28%	18.44%	9.69%
45_157	43	1	1	1	0	6.98%	3.49%	50	0	0	0	0	0.00%	0.00%	19	2	2	1	0	26.32%	11.84%	11.10%	5.11%
45_23	0	0	0	0	0	0.00%	0.00%	53	0	1	0	0	1.89%	0.94%	40	0	0	0	0	0.00%	0.00%	0.63%	0.31%
45_53	20	0	0	0	0	0.00%	0.00%	38	1	1	3	0	13.16%	7.89%	30	0	0	0	0	0.00%	0.00%	4.39%	2.63%
4_6	34	0	0	0	0	0.00%	0.00%	58	0	0	0	0	0.00%	0.00%	36	0	0	0	0	0.00%	0.00%	0.00%	0.00%
46_112	7	0	0	0	0	0.00%	0.00%	17	0	0	0	0	0.00%	0.00%	5	0	0	0	0	0.00%	0.00%	0.00%	0.00%
46_142	19	1	1	0	0	10.53%	3.95%	56	0	2	0	0	3.57%	1.79%	40	0	0	0	0	0.00%	0.00%	4.70%	1.91%
47_59	24	0	0	0	0	0.00%	0.00%	15	0	0	0	0	0.00%	0.00%	26	0	1	2	0	11.54%	7.69%	3.85%	2.56%

4_8	45	1<br>2	0	3	3	40.00%	18.33%	27	3	3		3	33.33%	19.44%	59	15	6	3	4	47.46%	22.03%	40.26%	19.93%
48_135	25	3	0	0	0	12.00%	3.00%	5	0	0	0	0	0.00%	0.00%	14	1	0	1	0	14.29%	7.14%	8.76%	3.38%
48_198	38	0	0	0	0	0.00%	0.00%	28	0	0	0	0	0.00%	0.00%	20	0	0	0	0	0.00%	0.00%	0.00%	0.00%
4_96	14	0	3	0	0	21.43%	10.71%	12	0	1	0	0	8.33%	4.17%	5	0	0	0	0	0.00%	0.00%	9.92%	4.96%
50_125	29	0	0	0	0	0.00%	0.00%	16	0	2	0	0	12.50%	6.25%	15	0	0	0	0	0.00%	0.00%	4.17%	2.08%
50_148	57	1	0	0	0	1.75%	0.44%	22	1	2	0	0	13.64%	5.68%	44	0	0	0	0	0.00%	0.00%	5.13%	2.04%
50_174	14	0	0	0	0	0.00%	0.00%	0	0	0	0	0	0.00%	0.00%	0	0	0	0	0	0.00%	0.00%	0.00%	0.00%
5_103	14	0	2	1	1	28.57%	19.64%	39	0	0	0	0	0.00%	0.00%	2	0	0	2	0	100.00%	75.00%	42.86%	31.55%
5_12	20	0	4	0	0	20.00%	10.00%	0	0	0	0	0	0.00%	0.00%	0	0	0	0	0	0.00%	0.00%	6.67%	3.33%
5_121	14	0	0	1	0	7.14%	5.36%	31	0	3	4	0	22.58%	14.52%	0	0	0	0	0	0.00%	0.00%	9.91%	6.62%
5_130	8	0	0	0	0	0.00%	0.00%	6	0	3	1	0	66.67%	37.50%	12	0	0	0	0	0.00%	0.00%	22.22%	12.50%
52_174	44	0	0	1	0	2.27%	1.70%	18	0	0	0	0	0.00%	0.00%	0	0	0	0	0	0.00%	0.00%	0.76%	0.57%
5_28	31	0	0	0	0	0.00%	0.00%	26	0	0	1	1	7.69%	6.73%	17	0	0	1	0	5.88%	4.41%	4.52%	3.71%
54_199	14	0	1	2	0	21.43%	14.29%	22	0	0	0	0	0.00%	0.00%	29	0	0	0	0	0.00%	0.00%	7.14%	4.76%
55_166	30	0	2	0	0	6.67%	3.33%	15	0	1	2	0	20.00%	13.33%	35	0	0	1	0	2.86%	2.14%	9.84%	6.27%
55_53	27	0	1	3	1	18.52%	13.89%	33	1	4	2	0	21.21%	11.36%	11	0	0	1	0	9.09%	6.82%	16.27%	10.69%
56_59	3	0	0	0	0	0.00%	0.00%	4	0	0	0	0	0.00%	0.00%	0	0	0	0	0	0.00%	0.00%	0.00%	0.00%
5_7	20	1	0	1	1	15.00%	10.00%	43	0	0	0	0	0.00%	0.00%	21	1	2	3	2	38.10%	26.19%	17.70%	12.06%
6101	21	0	0	0	0	0.00%	0.00%	0	0	0	0	0	0.00%	0.00%	0	0	0	0	0	0.00%	0.00%	0.00%	0.00%
6_107	37	1	0	0	0	2.70%	0.68%	0	0	0	0	0	0.00%	0.00%	0	0	0	0	0	0.00%	0.00%	0.90%	0.23%
61_93	0	0	1	0	0	0.00%	0.00%	44	2	3	4	1	22.73%	13.64%	17	4	4	2	0	58.82%	26.47%	27.18%	13.37%
6_3	13	0	2	0	0	15.38%	7.69%	13	0	0	0	0	0.00%	0.00%	13	0	0	0	0	0.00%	0.00%	5.13%	2.56%
63_192	13	0	2	0	0	15.38%	7.69%	18	0	0	0	0	0.00%	0.00%	16	0	0	0	0	0.00%	0.00%	5.13%	2.56%
6_32	22	1	2	2	2	31.82%	21.59%	35	6	6	0	2	40.00%	18.57%	20	3	4	2	1	50.00%	26.25%	40.60%	22.14%
6_36	15	0	1	2	2	33.33%	26.67%	29	2	1	5	1	31.03%	19.83%	3	0	0	1	0	33.33%	25.00%	32.56%	23.83%

64_96	0	0	0	0	0	0.00%	0.00%	0	0	0	0	0	0.00%	0.00%	0	0	0	0	0	0.00%	0.00%	0.00%	0.00%
65_77	13	0	0	1	0	7.69%	5.77%	5	0	0	0	0	0.00%	0.00%	16	0	0	0	0	0.00%	0.00%	2.56%	1.92%
66_40	14	0	0	1	1	14.29%	12.50%	40	0	0	0	0	0.00%	0.00%	24	0	1	0	0	4.17%	2.08%	6.15%	4.86%
66_71	78	1	0	5	0	7.69%	5.13%	40	0	3	0	0	7.50%	3.75%	32	0	0	0	0	0.00%	0.00%	5.06%	2.96%
6_75	31	0	0	0	0	0.00%	0.00%	42	0	0	0	0	0.00%	0.00%	29	0	0	0	0	0.00%	0.00%	0.00%	0.00%
68_111	10	0	0	0	0	0.00%	0.00%	14	0	5	0	0	35.71%	17.86%	7	0	0	0	0	0.00%	0.00%	11.90%	5.95%
6_86	54	3	1	11	2	31.48%	21.30%	28	1	1	7	3	42.86%	32.14%	36	4	7	8	6	69.44%	45.83%	47.93%	33.09%
69_55	20	0	0	0	0	0.00%	0.00%	64	0	3	0	0	4.69%	2.34%	35	0	1	1	0	5.71%	3.57%	3.47%	1.97%
70_2	2	0	0	0	0	0.00%	0.00%	0	0	0	0	0	0.00%	0.00%	1	0	0	0	0	0.00%	0.00%	0.00%	0.00%
7_1	17	0	1	1	2	23.53%	19.12%	0	0	0	0	0	0.00%	0.00%	0	0	0	0	0	0.00%	0.00%	7.84%	6.37%
7_108	26	1	2	0	0	11.54%	4.81%	32	0	3	0	1	12.50%	7.81%	0	0	0	0	0	0.00%	0.00%	8.01%	4.21%
7_109	9	0	0	0	0	0.00%	0.00%	15	0	0	0	0	0.00%	0.00%	21	0	0	0	0	0.00%	0.00%	0.00%	0.00%
7_13	0	0	0	0	0	0.00%	0.00%	0	0	0	0	0	0.00%	0.00%	0	0	0	0	0	0.00%	0.00%	0.00%	0.00%
7_130	33	0	0	0	1	3.03%	3.03%	26	0	0	0	0	0.00%	0.00%	0	0	0	0	0	0.00%	0.00%	1.01%	1.01%
7_133	47	0	0	0	0	0.00%	0.00%	9	0	4	2	0	66.67%	38.89%	0	0	0	0	0	0.00%	0.00%	22.22%	12.96%
7_136	3	0	0	0	0	0.00%	0.00%	2	0	0	0	0	0.00%	0.00%	26	0	0	0	0	0.00%	0.00%	0.00%	0.00%
71_67	2	0	0	0	0	0.00%	0.00%	7	0	0	0	0	0.00%	0.00%	26	0	0	0	0	0.00%	0.00%	0.00%	0.00%
7_188	7	0	0	0	0	0.00%	0.00%	15	0	0	3	0	20.00%	15.00%	17	2	3	0	0	29.41%	11.76%	16.47%	8.92%
73_173	0	0	0	0	0	0.00%	0.00%	0	0	0	0	0	0.00%	0.00%	0	0	0	0	0	0.00%	0.00%	0.00%	0.00%
73_193	48	0	0	0	0	0.00%	0.00%	23	0	0	0	0	0.00%	0.00%	27	0	2	1	0	11.11%	6.48%	3.70%	2.16%
74_20	52	0	1	0	0	1.92%	0.96%	30	0	0	1	0	3.33%	2.50%	0	0	0	0	0	0.00%	0.00%	1.75%	1.15%
74_25	38	0	0	0	0	0.00%	0.00%	21	0	2	0	0	9.52%	4.76%	34	0	0	0	0	0.00%	0.00%	3.17%	1.59%
7_45	20	0	0	2	1	15.00%	12.50%	4	0	0	1	0	25.00%	18.75%	41	2	4	4	1	26.83%	15.85%	22.28%	15.70%
74_86	35	1	3	4	0	22.86%	13.57%	33	1	3	4	0	24.24%	14.39%	25	3	3	8	1	60.00%	37.00%	35.70%	21.66%
75_140	48	0	0	0	0	0.00%	0.00%	50	0	0	0	0	0.00%	0.00%	32	0	1	0	0	3.13%	1.56%	1.04%	0.52%
75_156	32	0	0	0	0	0.00%	0.00%	27	0	0	0	0	0.00%	0.00%	28	0	0	3	0	10.71%	8.04%	3.57%	2.68%

7_54	50	0	0	0	0	0.00%	0.00%	66	0	0	0	0	0.00%	0.00%	15	0	0	0	0	0.00%	0.00%	0.00%	0.00%
76_100	7	0	0	0	0	0.00%	0.00%	0	0	0	0	0	0.00%	0.00%	0	0	0	0	0	0.00%	0.00%	0.00%	0.00%
7_73	34	0	2	1	2	14.71%	11.03%	16	0	0	0	0	0.00%	0.00%	33	4	6	8	2	60.61%	36.36%	25.10%	15.80%
78_109	35	0	0	0	0	0.00%	0.00%	53	0	0	0	0	0.00%	0.00%	29	0	0	0	0	0.00%	0.00%	0.00%	0.00%
78_2	11	0	1	0	0	9.09%	4.55%	8	0	0	0	0	0.00%	0.00%	18	0	0	1	1	11.11%	9.72%	6.73%	4.76%
78_5	16	0	0	0	0	0.00%	0.00%	0	0	0	0	0	0.00%	0.00%	0	0	0	0	0	0.00%	0.00%	0.00%	0.00%
79_146	12	0	0	0	0	0.00%	0.00%	16	0	0	0	0	0.00%	0.00%	25	0	0	0	0	0.00%	0.00%	0.00%	0.00%
79_198	54	9	3	5	3	37.04%	19.44%	44	10	2	5	2	43.18%	21.02%	19	1	3	1	2	36.84%	23.68%	39.02%	21.38%
79_40	46	0	0	0	0	0.00%	0.00%	33	0	0	0	0	0.00%	0.00%	35	0	0	0	0	0.00%	0.00%	0.00%	0.00%
8_115	21	0	0	0	0	0.00%	0.00%	25	0	0	0	0	0.00%	0.00%	30	0	1	0	0	3.33%	1.67%	1.11%	0.56%
8_146	29	1	0	0	0	3.57%	0.89%	55	0	0	0	0	0.00%	0.00%	28	0	0	0	0	0.00%	0.00%	1.19%	0.30%
8_148	36	2	2	13	6	63.89%	47.92%	23	0	3	3	0	26.09%	16.30%	15	1	0	3	0	26.67%	16.67%	38.88%	26.96%
8_17	33	0	0	0	0	0.00%	0.00%	19	0	0	0	0	0.00%	0.00%	5	0	0	0	0	0.00%	0.00%	0.00%	0.00%
8_20	21	0	2	2	0	19.05%	11.90%	8	1	1	0	0	25.00%	9.38%	36	0	1	0	0	2.78%	1.39%	15.61%	7.56%
8_39	30	0	2	5	2	30.00%	22.50%	10	0	0	1	0	10.00%	7.50%	25	0	0	0	0	0.00%	0.00%	13.33%	10.00%
8_40	43	1<br>2	6	3	1	51.16%	21.51%	42		7	2	3	28.57%	19.05%	31	3	2	5	1	35.48%	20.97%	38.40%	20.51%
8_57	17	0	0	0	0	0.00%	0.00%	0	0	0	0	0	0.00%	0.00%	0	0	0	0	0	0.00%	0.00%	0.00%	0.00%
8_66	44	5	7	2	3	38.64%	21.02%	45	7	3	4	3	37.78%	20.56%	28	1	1	3	2	25.00%	17.86%	33.8%	25.55%
8_81	51	5	2	1	0	15.69%	5.88%	28	0	0	0	0	0.00%	0.00%	0	0	0	0	0	0.00%	0.00%	5.23%	1.96%
8_92	23	0	1	1	0	8.70%	5.43%	34	0	2	4	0	17.65%	11.76%	12	0	0	0	0	0.00%	0.00%	8.78%	5.73%
8_99	20	0	1	0	0	5.00%	2.50%	43	0	3	0	0	6.98%	3.49%	7	0	0	0	0	0.00%	0.00%	3.99%	2.00%
9_130	19	1	0	0	0	5.26%	1.32%	17	1	0	0	1	11.76%	7.35%	24	0	0	0	0	0.00%	0.00%	5.68%	2.89%
9_145	63	0	0	0	0	0.00%	0.00%	36	0	0	0	0	0.00%	0.00%	55	0	0	0	0	0.00%	0.00%	0.00%	0.00%
9_157	26	0	0	0	0	0.00%	0.00%	29	0	0	1	0	3.45%	2.59%	35	0	2	0	0	5.71%	2.86%	3.05%	1.81%
ROC25	24	0	0	0	0	0.00%	0.00%	0	0	0	0	0	0.00%	0.00%	0	0	0	0	0	0.00%	0.00%	0.00%	0.00%

Yunzhe 89_7	31	0	0	0	0	0.00%	0.00%	24	0	0	0	0	0.00%	0.00%	0	0	0	0	0	0.00%	0.00%	0.00%	0.00%

Table S3 RT-qPCR primer sequence

Name	Sequence
Soff.05G0011330-4E _qF	GCCTCCAGAACTAGACAACCGAC
Soff.05G0011330-4E _qR	CAGTTGCCATCTCTTCGAACCC
Soff.04G0004430-2B _qF	GCTTGGAAAATTGATTCGGCTC
*Soff.04G0004430-2B*_qR	CGTCCCAACTTATCTCTAGCTC
*Soff.05G0002960-1B*_qF	ATGAAAGGCAGCTCATCAACGA
*Soff.05G0002960-1B*_qR	ACACTGTTGCTCCCATATGTCA
*Sspon.05G0014720-1A*_qF	TTTTACTCACTACACGACACC
*Sspon.05G0014720-1A*_qR	CTATATCCCAATCTTCCTTACCAC
*Sspon.05G0039660-1D*_qF	TCTTTTCCATGTTTGCGGACC
*Sspon.05G0039660-1D*_qR	TGCCATCTAGGGACAAATACCTC
*Sspon.03G0008230-3C*_qF	ATGATGAACTGGAAGCAGACGAC
*Sspon.03G0008230-3C*_qR	ACAAGGCACTAACTGACACGAG
*Soff.01G0015700-3D*_qF	ATAATCAGGAGGCAGTAGACC
*Soff.01G0015700-3D*_qR	CTGCAACTGCACGATCAC
*Soff.02G0004840-6H*_qF	AGATCGTTAGGTTCTGATCGAC
*Soff.02G0004840-6H*_qR	TTCGAGAACAGCATAATAACACC
*Soff.01G0000550-3E*_qF	ATGATAATATCCCCGACGATTCCTC
*Soff.01G0000550-3E*_qR	TTGCTTCCCGGTTTCGTGAG
GAPDH-qF	CACGGCCACTGGAAGCA
GAPDH-qR	TCCTCAGGGTTCCTGATGCC

Table S4 Molecular marker primer sequences

Name	Sequence
*Soff.05G0007900-1A*_qF	TCCAGTGGGTCGCGAATCTA
*Soff.05G0007900-1A*_qR	TCCGATGTGACACCCCAAAA
*Sspon.07G0015590-3C*_qF	GACGCCTTGACCTGAGTATG
*Sspon.07G0015590-3C*_qR	ATAATGCACAATTCCTCGAGAGC

Table S5 The results of transcriptome data quality control

Group	Sample	Raw bases (G)	Clean bases (G)	Q20 base (%)	Q30 bases (%)	GC content (%)
parent material	ROC25	11.22	11.2	98.06	93.8	53.44
	yunzhe89_7	11.57	11.55	98.05	93.82	53.24

R	1_48	11.24	11.22	97.99	93.5	53.87
	10_108	11.75	11.72	97.88	93.18	52.48
	10_123	11.38	11.37	97.5	91.93	53.83
	20_81	14.37	14.34	97.66	92.61	54.02
	21_177	11.17	11.15	97.76	92.84	53.58
	23_166	11.5	11.47	98.25	94.35	52.16
	25_38	11.2	11.19	97.74	92.74	53
	25_81	11.62	11.61	97.61	92.27	53.71
	27_23	11.51	11.49	97.82	92.86	53.93
	28_151	11.58	11.56	97.15	90.73	53.09
	28_6	11.3	11.29	97.79	92.81	53.54
	3_126	11.63	11.62	97.7	92.55	54.22
	4_37	11.84	11.83	97.9	93.17	53.5
	5_107	12.28	12.18	98.39	94.75	52.34
	7_109	11.33	11.33	97.9	93.36	53.26
	6_75	11.16	11.14	97.63	92.37	53.57
	7_54	11.53	11.52	97.69	92.45	52.84
M_S	1_115	11.16	11.14	97.31	92.4	52.79
	1_29	11.18	11.16	98.16	94.15	53.35
	2_53	11.66	11.64	97.24	92.19	53.53
	1_58	11.65	11.64	97.7	92.51	53.05
	2_105	11.91	11.9	97.74	92.71	53.5
	2_60	11.17	11.16	97.95	93.48	53.66
	22_131	12.12	12.1	97.96	93.52	53.49
	23_194	11.98	11.96	96.81	91.21	53.84
	24_142	14.28	14.26	97.39	92.17	52.79
	24_165	12.13	12.1	96.82	91.02	53.46
	4_122	11.7	11.69	97.22	92.17	53.71
	27_102	10.63	10.61	97.01	91.55	53.11
	27_28	11.91	11.89	96.94	91.43	53.97
	27_30	11.78	11.77	97.14	97.14	53.97
	28_21	11.92	11.9	97.41	92.72	54.02
	28_61	11.6	11.59	97.84	93.03	53.82
	3_69	11.15	11.12	97.62	92.34	53.21
	3_81	11.44	11.42	97.54	92.23	53.38
	66_71	11.57	11.54	97.22	92.28	54.38
	61_93	13.27	13.24	97.07	91.14	52.86
	69_55	11.96	11.93	97.87	93.17	53.69
	7_133	11.22	11.2	97.01	97.01	53.44
	7_188	11.97	11.95	97.14	90.86	53.01
	74_20	11.18	11.16	97.17	92.03	53.25
	74_86	13.6	13.59	97.04	91.27	52.7
	75_156	11.3	11.28	98.24	94.36	52.78

M_S	8_146	11.58	11.56	98.04	93.8	53.99
	8_20	11.12	11.11	97.6	92.23	54.81
	8_69	11.58	11.53	97.42	92.8	53.14
	8_81	11.89	11.87	97.51	91.98	54.55
	8_92	11.59	11.57	96.8	91.16	53.37
	9_130	12.02	12	97.09	91.84	53.69
	34_103	11.77	11.76	97.62	92.45	53.06
	34_71	11.58	11.55	97.39	92.76	54.85
	34_83	12.9	12.87	98.07	93.89	53.07
	35_148	11.36	11.34	97.55	92.12	53.89
	39_185	11.8	11.74	97.26	92.36	53.72
	4_122	11.7	11.69	97.22	92.17	53.71
	4_157	13.58	13.46	97.64	92.88	54.74
	4_158	13.05	13.03	97.4	92.27	53.19
	43_34	11.6	11.58	96.92	91.4	54.21
	45_157	11.88	11.86	97.89	93.21	53.43
	45_53	10.28	10.25	96.88	91.26	53.77
	48_135	14.39	14.36	97.75	92.83	53.74
	5_28	12.08	12.06	97.21	92.28	54.06
	5_7	11.71	11.69	97.48	92.05	52.88
	50_148	11.46	11.44	97.04	91.7	53.25
M_H	1_115H	12.7	12.66	97.95	93.58	54.04
	1_29H	11.34	11.32	98.12	93.85	53.99
	2_53H	11.66	11.64	97.2	92.19	53.53
	1_58H	13.88	13.84	97.48	92.19	53.62
	2_105H	11.13	11.12	97.82	93	53.18
	2_60H	11.6	11.59	97.93	93.37	53.63
	22_131H	11.34	11.32	97.78	92.76	53.19
	23_194H	11.6	11.58	97.14	92.03	53.21
	24_142H	11.44	11.42	97.07	91.97	52.92
	24_165H	11.9	11.89	96.8	91.11	54.07
	26_122H	11.21	11.19	97.04	91.68	53.68
	27_102H	13.74	13.74	97.28	91.44	53.18
	27_28H	10.62	10.6	97.42	92.7	52.75
	27_30H	11.41	11.39	96.91	91.3	53.22
	28_21H	11.05	11.03	97.74	92.68	53.81
	28_61H	11.1	11.08	97.98	93.43	53.33
	3_69H	11.36	11.34	97.81	93.03	53.56
	3_81H	11.65	11.64	97.98	93.51	52.67
	66_71H	11.17	11.16	97.74	92.6	53.81
	61_93H	11.63	11.61	96.85	91.15	52.98
	69_55H	11.61	11.59	98.05	93.73	53.08
	7_133H	11.48	11.45	97.07	91.89	53.12
	7_188H	11.26	11.24	97.01	91.64	53.33

	74_20H	11.26	11.23	96.5	90.11	52.74
	74_86H	10.83	10.82	97.09	91.82	53.47
	75_156H	11.49	11.48	97.86	93.14	53.87
	8_146H	11.83	11.81	97.79	92.89	53.38
	8_20H	13.49	13.46	97.79	92.93	53.96
	8_69H	11.63	11.63	97.06	91.79	53.14
	8_81H	12.08	12.06	97.53	91.98	52.64
	8_92H	11.34	11.33	96.93	91.38	54.16
	9_130H	15.58	15.55	97.1	91.26	52.78
	34_103H	12.07	12.05	97.85	93.1	53.57
	34_71H	11.41	11.39	97.14	91.98	54.19
	34_83H	11.48	11.47	97.84	93.09	52.84
	35_148H	11.5	11.48	97.75	92.79	53.72
	39_185H	11.97	11.95	96.87	91.31	53.83
	4_122H	11.06	11.04	96.95	91.43	53.71
M_H	4_157H	11.2	11.19	96.98	91.59	53.69
	4_158H	14.13	14.1	97.95	93.64	54.36
	43_34H	10.17	10.16	96.8	90.98	53.69
	45_157H	14.63	14.6	98.09	93.92	51.71
	45_53H	14.21	14.21	96.66	90.16	53.19
	48_135H	11.17	11.15	96.67	90.76	53.39
	5_28H	11.58	11.56	97.03	91.63	53.1
	5_7H	11.65	11.57	98.28	94.5	52.87
	50_148H	10.77	10.74	97.11	91.99	55.42
	28_31	12.08	12.05	98.23	94.41	53.36
	1_78	11.5	11.49	97.77	92.8	54.15
	1_92	11.58	11.56	97.82	92.88	53.4
	1_126	11.4	11.38	96.73	90.86	53.57
	3_120	11.09	11.08	97.63	92.41	53.06
	3_143	11.56	11.55	97.45	91.75	53.79
	3_77	11.91	11.88	97.26	91.4	53.72
	30_169	11.71	11.66	97.97	93.51	53.62
	4_166	11.41	11.4	96.93	91.35	52.93
S_S	4_28	11.54	11.52	97.79	92.91	53.93
	4_8	11.77	11.75	97.81	93.05	54.02
	5_103	11.45	11.44	97.72	92.78	53.9
	6_32	14.62	14.57	98.19	94.39	53.13
	6_36	12.09	12.07	97.84	92.92	53.38
	6_86	12.95	12.84	97.92	93.67	52.73
	7_45	11.86	11.84	96.94	91.47	53.85
	8_40	11.5	11.48	97.75	92.77	53.51
	8_66	11.63	11.61	97.74	92.81	54.41
	79_198	11.95	11.93	97.07	91.89	54.16

	36_105	11.64	11.62	96.92	91.44	54.38
S_H	28_31H	11.1	11.08	97.98	93.43	53.33
	1_78H	11.39	11.38	97.87	93.1	53.4
	1_92H	12.51	12.48	97.92	93.48	53.63
	1_126H	11.16	11.15	96.63	90.72	54.02
	3_120H	11.34	11.34	97.7	92.61	52.51
	3_143H	11.85	11.83	97.7	92.54	53.8
	3_77H	11.45	11.44	97.75	92.78	53.8
	30_169H	11.85	11.84	97.92	93.28	53.36
	4_166H	11.68	11.66	97.3	92.41	53.33
S_H	4_28H	11.75	11.73	97.96	93.42	53.23
	4_8H	11.67	11.66	97.57	92	52.77
	5_103H	11.94	11.93	97.6	92.4	54.54
	6_32H	11.25	11.24	97.59	92.18	54.05
	6_36H	11.91	11.9	97.58	92.21	53.29
	6_86H	11.19	11.17	97.31	92.45	52.63
	7_45H	11.88	11.87	96.98	91.53	52.69
	8_40H	11.69	11.65	98.1	93.88	53.96
	8_66H	11.52	11.5	97.9	93.32	54.13
	79_198H	11.15	11.14	96.89	91.38	53.7
	36_105H	12.06	12.04	97.06	91.76	54.1

Table S6 Enrichment of genes in the MAPK signaling pathway in plant

Gene_id	Gene/Protein	Description
Soff.00034750	LRR receptor-like serine	Participation in the defense against
Soff.00041170	WRKY24	Resisting rice blast
Soff.00066690	Chitinase	Chitinase
Soff.01G0008800-1B	Chitinase	Chitinase
Soff.01G0009880-1B	PYL4	Mediates plant ABA signal transduction
Soff.01G0014590-1A	Caltractin	Chitinase
Soff.03G0005540-1B	Probable Protein Phosphatase 2C (PP2C)	Plant signal transduction

Soff.04G0005960-2C	PYL8	Mediates plant ABA signal transduction
Soff.09G0002280-1C	Mitogen-Activated Protein Kinase Kinase Kinase (MAPKKK)	MAPK3
Soff.10G0010870-3F	Chitinase	Chitinase
Soff.10G0010910-2B	Chitinase	Chitinase
Sspon.01G0008670-2P	EIN3	Ethylene signal transduction
Sspon.02G0018840-4D	CTR1	Negative regulation of ethylene
Sspon.03G0008230-3C	Mitogen-Activated Protein Kinase Kinase Kinase (MAPKKK)	MAPK3
Sspon.07G0002940-4D	Mitogen-Activated Protein Kinase Kinase Kinase (MAPKKK)	MAPK3
Sspon.07G0003190-1A	Reversion-to-Ethylene Sensitivity 1（RTE1）	Regulating ethylene
Sspon.07G0030050-1C	WRKY22	Involvement in pathogen defense, mediating leaf senescence

Table S7 The DEGs enriched in biological processes in response to stimulus

Group	Gene name	Gene/Protein	Description

S1 & M1	*Soff.01G0011110-3D*	TIFY	Plant development and defense
	Soff.03G0004270-1P	Aspertic Protease in Guard Cell 1 （ASPG1）	Cell senescence, stress, programmed death
	Soff.04G0003910-1A	WRKY1	Plant ABA signal transduction
	Sspon.01G0016030-3D	RGA2	Plant defense
	Sspon.02G0022580-3C	RPM1	Plant immune receptor protein
	Sspon.05G0018560-2C	RGA2	Plant defense
S4 & M4	*Soff.02G0001540-4E*	O-methyltransferase 2	Plant defense
	Soff.05G0002960-1B	PIK6	Plant defense
	Soff.06G0000250-1E	ERF115	Related to plant regeneration
	Sspon.01G0037110-1B	Thaumatin-Like Protein 1b （TLP）	Participate in host defense process
	Sspon.03G0026370-1B	Lateral Root Primordium 1 （LRP）	Lateralroot primordia

Table S8 The KEGG enrichment analysis of Hub genes

Phenotypic traits	Module （+/-）	Pathway	*P*-value
R	R-green（-）	Nitrogen metabolism	0.006198836
		Glycosaminoglycan degradation	0.007646526

M_S	M_S-yellow（+）	ABC transporters	0.0354888
		Galactose metabolism	0.03839617
M_H	M_H-yellow（-）	Phenylpropanoid biosynthesis	0.078219
S_S	S_S-blue（+）	MAPK signaling pathway - plant	0.002878329
		Phosphatidylinositol signaling system	0.03645857
	S_S-turquoise（+）	MAPK signaling pathway - plant	0.008604351
		Amino sugar and nucleotide sugar metabolism	0.009075563
		Plant-pathogen interaction	0.02573205
	S_S-yellow（+）	ABC transporters	0.0354888
	S_S-green（+）	Nitrogen metabolism	0.01848234
		Butanoate metabolism	0.01960359
		Galactose metabolism	0.03839617
		Glyoxylate and dicarboxylate metabolism	0.04350447
S_H	S_H-yellow（-）	Glycosaminoglycan degradation	0.005104082
		ABC transporters	0.02380083

Note: Only biological pathways with *P*-value < 0.05 were selected; (+): Positive correlation; (-): Negative correlation

Table S9 The GO enrichment analysis of Hub genes

Phenotypic traits	Module	Biological pathway	*P*-value

R	R-brown（+）	Defense response	0.0001
		Response to stress	0.001368
		Response to stimulus	0.003942
		Nitrate transport	0.009697
		Threonyl-tRNA aminoacylation	0.010083
	R-green（-）	Cellular response to nitrate	0.002198
		Cellular response to reactive nitrogen species	0.002198
		Tetrahydrofolate metabolic process	0.002564
		Folic acid-containing compound metabolic process	0.00357
		Pteridine-containing compound metabolic process	0.00421
M_S	M_S-yellow（+）	Seed coat development	0.000176
M_H	M_H-yellow（-）	Seed development	0.00367
		Fruit development	0.005024
		Hyperosmotic response	0.007287
		Hyperosmotic salinity response	0.007287
		Reproductive structure development	0.029977
S_S	S_S-blue（+）	Auxin-activated signaling pathway	0.001895
		Cellular response to auxin stimulus	0.003137
		Trichome branching	0.006581
		Oxaloacetate transport	0.007401
		Simple leaf morphogenesis	0.009038
	S_S-turquoise（+）	Seed germination	9.17E-05
		ER to Golgi vesicle-mediated transport	0.000182

		Seedling development	0.00044
		Response to oxygen-containing compound	0.001125
		Response to acid chemical	0.002332
	S_S-yellow（+）	Negative regulation of microtubule polymerization or depolymerization	0.001832
		Negative regulation of microtubule polymerization	0.001832
		Negative regulation of protein complex assembly	0.001832
		Negative regulation of protein polymerization	0.001832
		Negative regulation of cytoskeleton organization	0.001832
	S_S-green（+）	Response to inorganic substance	0.001095
		Cellular response to nitrate	0.002747
		Cellular response to reactive nitrogen species	0.002747
		Tetrahydrofolate metabolic process	0.003204
		Folic acid-containing compound metabolic process	0.004461
S_H	S_H_yellow（-）	Seed coat development	1.94E-05
		Seed development	0.000416
		Fruit development	0.000574
		Negative regulation of microtubule polymerization or depolymerization	0.000733
		Negative regulation of microtubule polymerization	0.000733

Note: Only the top five biological processes with the smallest *P*-value values were selected; (+): Positive correlation; (-): negative correlation

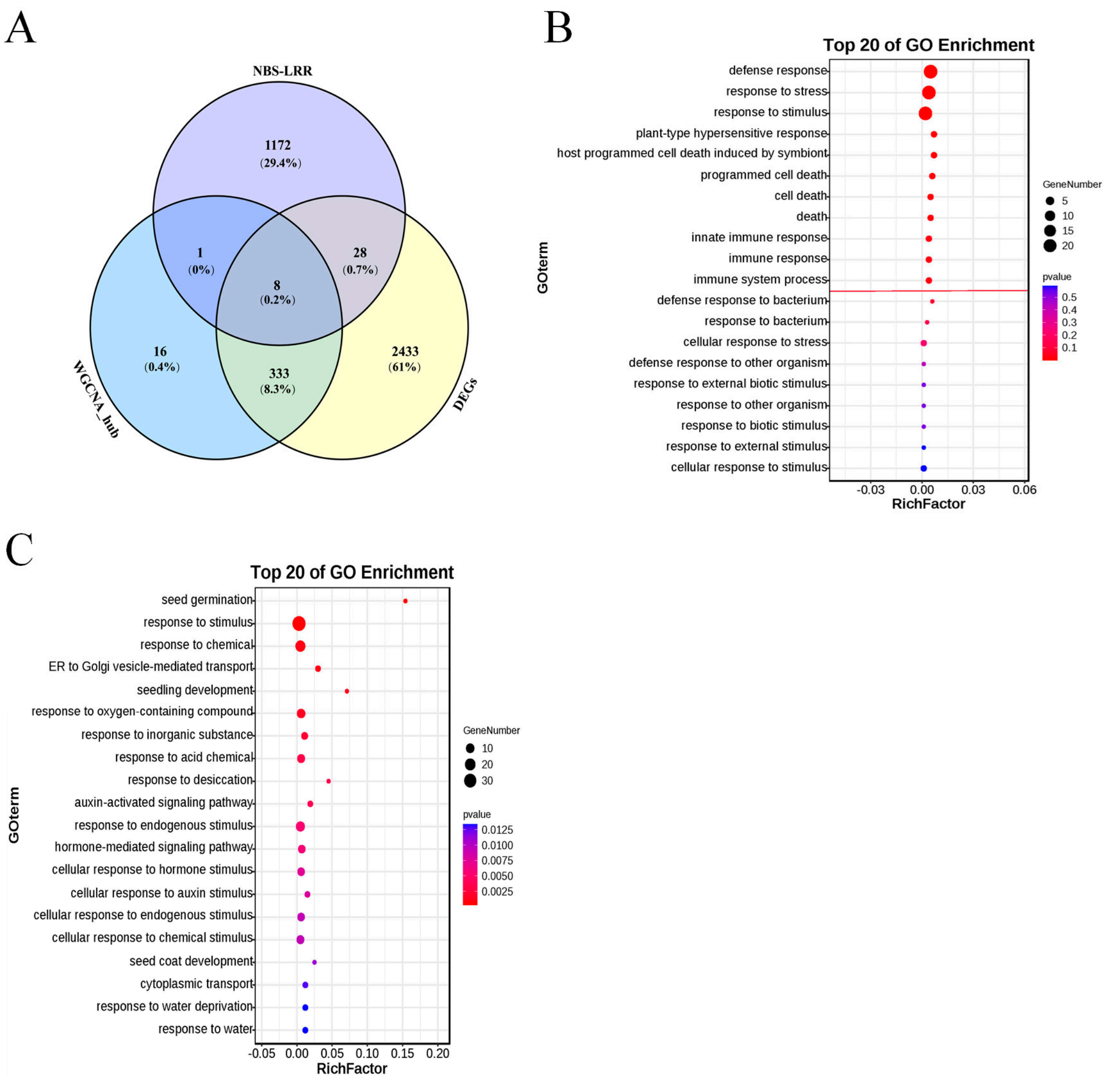

Figure S1 Identification and functional enrichment of key regulatory and disease resistance genes. (A) WGCNA _Hub & NBS-LRR & DEGs Venn-Analyse; (B) GO enrichment analysis of DEGs and NBS-LRR crossover genes, above the red line are the pathways with *P*-value <0.05; (C) GO enrichment analysis of DEGs and WGCNA_Hub_gene intersection genes
